# Adapting to the algorithm: how accuracy comparisons promote the use of a decision aid

**DOI:** 10.1186/s41235-022-00364-y

**Published:** 2022-02-08

**Authors:** Garston Liang, Jennifer F. Sloane, Christopher Donkin, Ben R. Newell

**Affiliations:** School of Psychology, The University of New South Wales, Sydney, Kensington, NSW 2052 Australia

**Keywords:** Algorithm, Decision aid, Feedback

## Abstract

In three experiments, we sought to understand when and why people use an algorithm decision aid. Distinct from recent approaches, we explicitly enumerate the algorithm’s accuracy while also providing summary feedback and training that allowed participants to assess their own skills. Our results highlight that such direct performance comparisons between the algorithm and the individual encourages a strategy of selective reliance on the decision aid; individuals ignored the algorithm when the task was easier and relied on the algorithm when the task was harder. Our systematic investigation of summary feedback, training experience, and strategy hint manipulations shows that further opportunities to learn about the algorithm encourage not only increased reliance on the algorithm but also engagement in experimentation and verification of its recommendations. Together, our findings emphasize the decision-maker’s capacity to learn about the algorithm providing insights for how we can improve the use of decision aids.

**Supplementary Information:**

The online version contains supplementary material available at 10.1186/s41235-022-00364-y.

## Introduction

Decision aids are increasingly in demand. Often implemented as computer algorithms, developments in data availability and computational capabilities have expanded the reach of these tools into much of everyday life. From the mundane, such as deciding which TV show to binge next, to the momentous, such as recommending surgery to a patient, algorithms synthesize vast amounts of information to provide users with on-demand recommendations.^1^

The focus of this paper is to understand what guides individuals to rely upon a recommendation rather than making their own decision. Decision aids, while powerful, might not be the panacea to every problem. The uncertain surgeon who seeks out a medical decision aid by day might later ignore the algorithm behind Netflix’s show recommendations by night.

In this paper, three experiments show that individuals exhibit an acute selectivity in when they rely upon a recommendation. Across our experiments, we instantiate an imperfect but helpful algorithm into a perceptual decision-making task. We show that the information individuals learn about the accuracy of an algorithm is crucial to when individuals rely on a recommendation. Understanding one’s relative performance compared to the algorithm’s accuracy equips the decision-maker with the knowledge of who (or what) is better suited to solving the problem at hand. Taken together, we undertake a systematic comparison of feedback, training, and strategic hints to understand how learning about the algorithm affects the way people use recommendations.

### Comparing algorithm performance

There is a notable distinction between seeking advice from another person compared to seeking an algorithm’s recommendation. For a person, the decision-maker can put themselves in another’s shoes. The advisor may share the same reasoning process and step the person through the complexities of a situation (Prahl & Van Swol, 2017). By contrast, the steps an algorithm takes to produce a recommendation may be opaque or at the least unfamiliar to the ordinary user (Yeomans et al., 2019). To ameliorate this gap, algorithms are typically accompanied by descriptions that help convey why its recommendations can be trusted, for instance, by describing the mechanics of its statistical underpinnings. Such information can help decision-makers calibrate their expectations about how useful a recommendation might be.

A simple way to communicate a recommendation’s usefulness is to provide information about the algorithm’s *accuracy*. Accuracy highlights any performance benefits of relying on the recommendation and offers a benchmark against which individuals can judge their own performance (Parasuraman et al., 2000). Typically, accuracy is conveyed through (a) *verbal descriptions* that summarize performance, such as describing the algorithm as an 87% accurate medical diagnostician (e.g. in Longoni et al., 2019), or (b) *feedback* accumulated over multiple recommendations, such as providing information about what the algorithm recommended compared to the correct response (e.g. Dietvorst et al., 2015). In either format, accuracy information establishes a simple explanation for why a recommendation is or is not used; namely, that the preferred system (algorithm or personal judgement) is superior in performance.

Perhaps most interesting are instances where superior recommenders are shunned even in the presence of accuracy information promoting their virtues (e.g. Dietvorst et al, 2015; Mohoney & Houpt, 2019; Barlett & McCarley, 2017, 2019). A good example comes from a set of experiments involving feedback and a helpful decision rule (Arkes et al., 1986). Participants examined student report cards and based upon three grades were asked to indicate the honours-roll status of each student (i.e. responding honours/not honours after each report card). Additionally, they were provided with a simple decision rule to aid them. The rule was 70% accurate: indicate honours for report cards with two or more A’s, and no honours for one or fewer.

Various instruction manipulations made clear the difficulty of surpassing this performance benchmark. For example, the debias condition was explicitly instructed that “most people can’t judge at a rate better than 70% correct … [those] who try actually perform a lot worse” (Arkes et al., 1986, p. 97). However, despite the heavy-handed instructions and ongoing feedback throughout the task, most individuals deviated from exclusively using the decision rule and scored lower than had they strictly complied. Surprisingly, this rule deviation was more prominent when feedback was present than when it was absent.

These rather curious results suggest that many individuals believed they could outperform the rule. Such behaviour may have been driven by scepticism about the validity of the rule, participants’ belief that their prior knowledge of college grades was superior to a simple rule, insufficient training in the task, or perhaps simply the desire to take on the challenge implied by the experimenter (e.g. “I am superior to most people so I will be able to do better”). Whatever the precise motivation, these kinds of results highlight the importance of being able to accurately assess one’s own level of (unaided) performance on a task when deciding whether to seek and follow an external recommendation (Arkes et al., 1986; Sieck & Arkes, 2005).

### A matter of skill

Algorithmic decision aids hold a great deal of promise for highly skilled professions (e.g. sentencing decisions by judges; Kleinberg et al., 2018). Particularly in time-poor environments, algorithms can be helpful in outsourcing the peripheral features of a task and allowing the expert to focus on the more demanding details. Radiography is one such profession where visual search algorithms assist expert judgement in the detection of screening anomalies. Radiologists can outsource ambiguous cases to visual search algorithms that in turn recommend which anomalies require additional expert scrutiny.

Expertise is precisely what equips individuals to judge the utility of any decision aid tool. Expertise can also, however, be an impediment to using decision aids. Relative to lay populations, more knowledgeable experts typically reject recommendations from both algorithmic and human advisors (Logg et al., 2019; Yaniv, 2004; Arkes et al., 1986). Within the medical field, high levels of expertise typically beget overconfidence, where overestimating one’s own capabilities can lead to grave judgement errors (Berner & Graber, 2008; Croskerry & Norman, 2008; Sieck & Arkes, 2005).

Examining how people evaluate their skills relative to the algorithm can help determine when one should consult a decision aid. Our experiments incorporate manipulations that vary the complexity of training and veridical feedback to give people multiple opportunities to reassess their performance. Having multiple opportunities to re-evaluate their performance may lead individuals to adapt their reliance on a decision aid over time. For example, an individual may decrease their reliance if their skills gradually improve beyond the accuracy of the algorithm. However, if the algorithm consistently outperforms the individual, that individual may learn to be increasingly reliant on the algorithm’s suggestion.

Single-shot choice experiments have found that individuals adjust their preference for a decision aid based on information about its accuracy. For instance, while participants initially preferred a human physician to an equivalent-performing algorithm in a medical scenario, Bigman and Gray (2018) found a preference switch when participants were subsequently told the algorithm would outperform the physician. Individuals are also capable of disregarding unhelpful decision aids such as when they are told the recommendations are generated by a coin-flip (i.e. chance-level performance in a binary choice task; Douneva et al., 2019). Our experiments sought to combine and extend these findings in a within-subject investigation of how performance information alongside assessments of one’s own skill shapes when people consult an algorithm.

### Overview of experiments

**Fig. 1:**
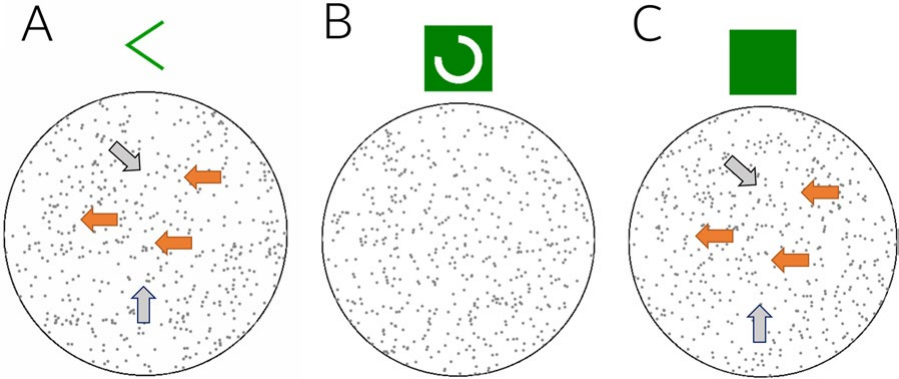
Examples of dot motion stimuli with the algorithm. In the task, a proportion of dots move along the 90°–270° axis coherently and participants judge the direction of dot movement along this axis as either left-motion or right-motion (shown in the orange arrows). Distractor dots move in straight lines but at different axes (shown in grey arrows). Note that the orange and grey arrows appear here only for illustrative purposes; they were never present for any participants in any experiment. Panel **A** shows the algorithm’s recommendation (left green arrow) above the stimulus. In Experiment 1a and 1b the recommendation appeared above the stimulus automatically. In Experiments 2 and 3 the algorithm appeared on screen as a green box (Panel **C**) unless the participant made a request for a recommendation. If requested, the algorithm loaded the recommendation during a one-second delay displaying a “loading circle” that revolved around the box (Panel **B**). All motion in the stimulus stopped during this loading time and then resumed once the recommendation was revealed (as in Panel **A**)

Across three experiments, we investigated how people relied on a decision aid that was situationally helpful. Figure 1 displays and describes the way in which we implemented the decision aid (see figure caption for details). In the main task, individuals made binary choice judgements that could be aided by an algorithm. If participants were uncertain, they could consult an algorithm that was set to a known accuracy level of 70%. This meant that on most, but crucially not all, occasions the algorithm would provide a correct recommendation (e.g. an arrow pointed in the recommended direction for the dot motion task, see Fig. 1). Importantly, participants were explicitly told of the algorithm’s accuracy level and the potential for an incorrect recommendation (i.e. 30% of the time the arrow points in the opposite direction to the motion of the dots).

We specifically chose the algorithm’s accuracy level to bisect the expected performance across two levels of task difficulty (explained further in Experiment 1a and 1b). For the easier stimuli, most individuals learnt the task to near perfection, and the vast majority surpassed the accuracy of the algorithm (i.e. median participant accuracy ~ 95% correct). By contrast, the harder versions of the stimuli continually proved to be difficult, even with increasing levels of training and feedback introduced in later experiments. The algorithm systematically outperformed all but a single individual for the harder version of the task (median participants’ accuracy ~ 52% correct).

Our primary aim was to examine how people subsequently adjusted their use of the 70%-accurate algorithm to these difficulty levels. A noteworthy implication of fixing the algorithm’s accuracy across stimulus difficulty is that the task includes situations where what is difficult for an algorithm may not be difficult for a human observer (i.e. easier trials where the algorithm is 70% correct). While we acknowledge this is not always the case, such situations can arise if the algorithm uses a different process compared to a human observer. For example, in a task distinguishing huskie dogs from wolves, a human may recognize the facial subtleties of each animal while an image classifier might learn to recognize snow in the background of images of wolves (Ribeiro et al., 2016). Indeed, online CAPTCHA tests exist because classifier algorithms have difficulty recognizing simple objects that humans can easily identify. Our intent in including such situations is that we can directly examine whether individuals understand such limitations of the algorithm.

An additional benefit to this experimental setup is that it discouraged the exclusive reliance on either source of responses. Should an individual display an inherent aversion to the algorithm, their performance for the harder images would be at chance levels. Similarly, an individual that outsourced the entirety of the task to the decision aid would make a substantial number of simple and avoidable errors on the easier images. The best overall approach was to selectively seek the algorithm’s recommendation for the harder stimuli but disregard its recommendation for the easier stimuli.

Examining a strategy of *selectively* using the algorithm distinguishes our experimental settings from many past studies where the best response is *always* to use the algorithm instead of one’s own judgement (Arkes et al., 1986; Dietvorst et al., 2015; Logg et al., 2019). While it is possible, via sufficient experience and feedback, that participants can learn that the best response strategy is always to rely on the algorithm (e.g. Sieck & Arkes, 2005), there is no guarantee that such a policy will be implemented. Repeated experience may instead inspire a variety of hypotheses regarding what behaviour is appropriate, such as wondering “does the experimenter always expect the same response or should I intervene across different stimuli?” (Brehmer, 1980), and, in turn, lead to maladaptive experimentation and suboptimal responding (Szollosi et al., 2019).

Our intent was to remove this experimental layering by including situations in which the best response was to *avoid* the decision aid (e.g. on an easier trial, participants may judge their own performance to be superior to a 70%-correct algorithm). These *avoid trials* provide the additional space for participants to exhibit their understanding of the task. By adjusting one’s reliance on an algorithm, our data allow for richer characterizations of people’s decision-aid behaviours beyond a dichotomy of algorithm users and avoiders.

## Experiment 1a and 1b: automatic recommendations

We begin with situations where recommendations are provided automatically and without cost to the decision-maker. Such automatic recommendations resemble alert systems that monitor data and only interrupt the decision-maker when a criterion is met (e.g. emergency ward alerts when patient vitals fall below critical thresholds). In Experiment 1a participants learnt to categorize mammogram images as cancerous or non-cancerous and in Experiment 1b, a separate group of participants performed the dot motion judgement task outlined in Fig. 1. In both experiments, participants were provided with recommendations from an algorithm described as being 70% accurate. Our key question was whether adherence to this recommendation differed as a function of the difficulty of the to-be-classified stimulus. We hypothesized that individuals would avoid relying on the decision aid for easier images and reserve its use for the harder images.

### Method

#### Participants

Experiment 1a and Experiment 1b were identical in design with only stimuli differences (see below). Experiment 1a was conducted with 55 psychology undergraduates (*M*_age_ = 19.1, SD = 1.16, female = 34) at UNSW, Sydney. Experiment 1b involved 32 participants drawn from the same pool (*M*_age_ = 19.1, SD = 1.16, female = 16). Participants received course credit for participation and were awarded a proportional payment out of $5.00 AUD based upon their performance in the task (*M*_1a_ = $3.44, SD_1a_ = 0.21, *M*_1b_ = $3.73, SD_1b_ = 0.22). Sample size was determined on the basis of past similar experiments of training in categorization (Giguère, & Love, 2013; *n* = 50) and dot motion with similarly large numbers of within-subject trials (e.g. Pilly & Seitz, 2009; *n* = 12).

#### Materials

##### Stimuli

Experiment 1a and 1b used different stimuli. Experiment 1a involved categorizing mammogram images as either cancerous or normal. We obtained anonymized images from the Digital Database for Screening Mammography (DDSM) that is freely available online (Heath et al., 2001).

To understand our results better using stimuli over which we had more experimental control, Experiment 1b used random dot arrays. These arrays were adapted from the native random dot motion plugin for JSPsych (de Leeuw, 2015; example in Fig. 1). In the array, 300 Gy dots move across the screen in various straight lines with a proportion of the dots coherently moving along the 90°–270° axis. The task requires participants to determine the direction of movement along this axis as either left-motion or right-motion (shown in the orange arrows in Fig. 1). Distractor dots moved in straight lines but along different axes (shown in grey arrows). The difficulty of the task was manipulated through the proportion of coherently moving dots. For example, a higher coherence level indicates a larger proportion of dots moving along the 90°–270° axis.

Prior to each experiment, we conducted pilot testing to determine the difficulty of the stimuli. In general, difficulty was determined based on the performance of pilot participants in two additional separate experiments (*N* = 107 for mammogram pilot, *N* = 34 for dot motion pilot). In these pilot experiments, participants were presented with the perceptual task and asked to categorize the stimuli to their best ability. Average levels of performance were determined for each individual image in the case of mammograms (hence the larger sample size) and each coherence level for the dot motion stimuli. In brief, stimuli for which performance was relatively high (i.e. ~ 80% correct for mammograms, ~ 90% correct for dot motion) were labelled “easier”, whereas stimuli for which performance was near chance levels (i.e. ~ 55% correct for both stimuli types) were labelled “harder”. For Experiment 1a, we retained 267 mammogram images from an initial sample of 471 images using the above performance criterion. For Experiment 1b and all subsequent experiments, we selected coherence levels of 0.25, 0.2, 0.02, and 0.01 where the former two levels were labelled “easier” and the latter two “harder”. The full details of these pilot experiments are presented in Additional file 1.

##### Decision aid algorithm

The algorithm was instantiated as a probabilistic cue that was positioned above the stimulus. In Experiment 1a, the algorithm’s recommendation was a red circle that signalled cancer-category membership. In Experiment 1b, the recommendation was a left-pointing arrow that signalled leftwards motion. This means the algorithm signals only a single outcome (cancer/left). This design feature was originally inspired by mammogram images where a decision-maker may prioritize identification of cancer positive outcomes rather than non-cancerous outcomes. While this asymmetry in the outcomes does not translate to random dot stimuli, we retained the single-outcome cue in order to facilitate comparisons between the experiments.

For trials when the recommendation appeared, its onset was simultaneous with the onset of the stimulus. Participants were told that when the recommendation appeared, the algorithm would signal the correct category on 70% of occasions. This performance constraint means that stimulus categories were unbalanced such that 70% of the cued images were *cancer*/left stimuli and 30% were *normal*/right stimuli. We refer to the algorithm’s recommendation as the *cue.*

The test stage was separated into cued blocks, when the algorithm appeared, and control blocks. In the cued blocks, the cue appeared on *half of the images* and for an equal number of easier/harder images. Presenting the cue for half the stimuli meant that the absence of the cue did not always indicate the image was a *normal*/right stimuli although it was more probable due to the unbalanced proportions of stimuli. We return to the interpretation of non-cued images in the “Discussion” section. Each participant received a random subset of images for which the cue would appear. In the control blocks, participants were reminded the cue would never appear before the block began. We included the control block to isolate the influence of the cue on responses (see Fig. 1).

##### Decision aid algorithm description

In the instructions and as a reminder at the start of each cued block participants were told, “The algorithm is there to help you—whenever you see the cue, there is a 70% chance that the *image* (dots in the panel) was a *cancer image* (moving to the left). Conversely, there is a 30% chance that the cue is indicating the incorrect response and the *image* (dots in the panel) is a *normal image* (moving to the right).” (Italics show instructions for Exp. 1a, instructions for Exp. 1b in parentheses). Participants were reminded that it was up to them to decide if they wished to use the cue or rely upon their own judgement.

#### Design

The experiments used a within-subject design where block type (cued and control block) alternated throughout the experiment. The first block was randomized between-subjects and collapsed in the analyses.

##### Training and test blocks

Both experiments were divided into an initial training stage followed by a longer test stage without feedback. In Experiment 1a, the number of trials in each stage was constrained by the number of unique mammograms from the norming procedure. Experiment 1b did not have these constraints as the random dot motion stimuli were computer generated. Consequently, the training stage of Experiment 1a consisted of 44 easier mammogram images (i.e. 22 easier cancer and 22 easier normal images). The training stage in Experiment 1b consisted of 80 easier images (40 left-motion and 40 right-motion). As a brief aside, our decision to train participants on easier images and then test them on a combination of harder and easier images follows from work on the impact of idealized training in category learning (Giguere & Love, 2013; Hornsby & Love, 2014).^2^

**Fig. 2:**
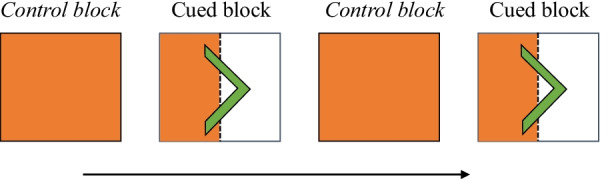
Test block progression in Experiment 1a and Experiment 1b. Difficulty of the test stimuli was randomized within each block of trials. White proportion denotes the proportion of trials when the arrow/cancer cue appeared. In the control block, the cue never appeared. In the cued block, the cue appeared on a random half of the block. Though the figure may appear to show cue-absent trials appeared in the first half of the block, the actual order of cued and cue-absent trials was randomized within the block

Each test block of images contained 80 images made up of the 2 × 2 category by difficulty matrix. Specifically, in Experiment 1a each block consisted of 20 easier cancer, 20 easier normal, 20 harder cancer, and 20 harder normal mammograms. There were four test blocks in total (for progression, see Fig. 2). Experiment 1b also consisted of the same 80-image matrix with left-motion or right-motion categories and a total of six test blocks.

#### Procedure

Participants were introduced to the categorization task and given examples of each stimulus category prior to starting their training. They were told that their task was to categorize their respective stimuli as either *cancer* (left-motion) or *normal* (right-motion). Participants entered their responses on a keyboard with the *cancer* (left-motion) response mapped to the “c” key and *normal* (right-motion) responses mapped to the “n” key. The instructions explained that in the training stage, they would receive feedback following each image informing them of the correct category. Feedback appeared below the stimulus as either green text for correct responses or red text for incorrect responses. Individuals entered responses to proceed to the next trial. A fixation cross was displayed for 1.5 s that separated the start of the following trial. In training, responses slower than 5 s were given feedback to speed up.

Following training,^3^ a new set of instructions then described the test stage and the algorithm (cue). In both experiments, participants were told the cue would appear above the stimulus and could help them by signalling the probable correct response. The algorithm description statement (see “Materials” section) was presented. Instructions then explained that the test stage would be separated into the two block types: *cued* blocks, where the cue would appear on a random half of the trials, and *control* blocks, where participants would complete the task on their own (see Fig. 2). A short quiz was administered prior to starting the test stage to ensure participant understanding of the instructions. Block type alternated throughout the task. Between each block a reminder screen stated either the cue’s chance of being correct (e.g. 70% chance of cancer) or a reminder that the upcoming control block would never display the cue. Once complete, participants were paid based upon their overall proportion of correct responses.

### Results

**Fig. 3:**
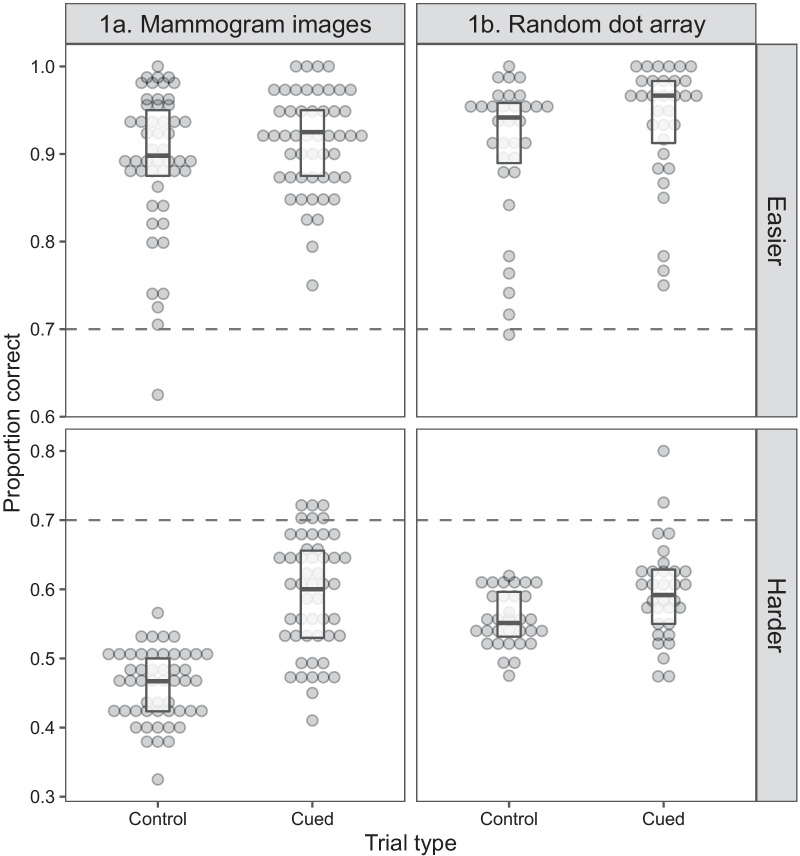
Proportion of correct responses during the test stage as a function of trial type and stimulus difficulty. Control trials, when the cue is never presented, are compared against cue-present trials in the cued block. Boxplots display the median and interquartile ranges with dots representing individual participants. Green intercept line represents the algorithm’s performance (i.e. 70% correct)

For this and following experiments, we report Bonferroni corrected p-values for analyses involving multiple comparisons and remove responses with extreme response times (slower than 10 s, 0.04% of trials; or faster than 0.18 s, 0.2% of trials). Recall that the test stage alternated between the control blocks and the cued blocks where the algorithm recommended one response (cancer in Exp. 1a, left-motion in Exp. 1b). In Fig. 3*,* we separately present these trial types in each experiment.

Beginning with the easier trials (top row of Fig. 3), the proportion of correct responses was high across both experiments (*M*_1a_ = 0.89, SD_1a_ = 0.08, *M*_1b_ = 0.92, SD_1b_ = 0.08). Nearly all individuals, except for a single participant in each experiment, surpassed the accuracy of the cue. To gauge the influence of the cue, we calculated difference scores in proportion correct between the cued trials and the control trials. In both experiments, the cue produced a minor numerical improvement (mammogram images, *M*_diff_ = 0.02; dot motion *M*_diff_ = 0.03). This high level of performance suggests that on the 30% of trials when the cue was misleading, individuals were able to overrule its recommendations.

The lower panels of Fig. 3 present performance in the harder trials. Overall performance was worse for the harder stimuli than the easier stimuli as indicated by a main effect of difficulty (*F* (1, 80) = 1182.20, *p* < 0.001, *η*_*p*_^2^ = 0.93). Difference scores between cued and control blocks showed larger improvements for the mammogram images in Exp. 1a (*M*_diff_ = 0.13, SD_diff_ = 0.07) as compared to the dot motion stimuli in Exp. 1b (*M*_diff_ = 0.04, SD_diff_ = 0.07). This difference was supported by a two-way ANOVA with a significant difficulty (hard vs easy) by experiment (1a vs 1b) interaction (*F*(1, 80) = 21.68, *p* < 0.001; *η*_*p*_^2^ = 0.21). Despite this improvement, most participants performed worse relative to the accuracy of the algorithm in the cued trials (algorithm’s accuracy = 0.70; *M*_1a_ = 0.59, SD = 0.08, *t*(49) = − 9.13, *p* < 0.001; *M*_1b_ = 0.60, SD = 0.07, *t*(31) = − 8.59, *p* < 0.001). This suggests that on occasion, participants also disagreed with the cue when it appeared. Together, our results show that participants selectively relied on the cue for the harder trials but could have improved their performance had they agreed with the algorithm’s recommendation more often.

### Discussion experiment 1a and 1b

Across both experiments, we found that individuals relied upon the algorithm’s recommendation for the harder stimuli and ignored the cue for easier stimuli when it was potentially misleading. For the harder images, participants improved their performance when the cue appeared by agreeing with the *cancer* (left-motion) recommendation. Curiously, participants also overruled the cue for the harder images on a minority of cued trials, presumably, to correct for the knowledge that there would be misleading recommendations. As an aside, we examined whether the overruling patterns resembled probability matching (e.g. responding “cancer” for 70% of the cued-images and “not-cancer” for the remaining 30%—see Additional file 1 for details). Although seemingly plausible in the aggregate, probability matching did not appear in the individual-level data.

While these initial results were encouraging, certain features of the cue in Experiment 1a and 1b limited our understanding of how participants recruited the recommendation. The first feature was that the cue always prompted a single response (cancer, left-motion). One problem this creates is determining whether participants inferred anything from the *absence* of the cue. It is possible participants interpreted this absence to signal the opposite of the cued response (normal or right-motion). Introducing a recommendation that can signal both responses would ameliorate this concern. Second, the fact that the cue appeared unpredictably obscured whether participants actually needed the recommendation for a particular stimulus. In the next experiment, we addressed both features by handing individuals control over when they sought out a recommendation.

An open question is whether people would overrule a recommendation that was sought out rather than automatically provided. Akin to the idea of sunk costs, overruling the algorithm may be unappealing given the effort to acquire the recommendation in the first place, and especially if the participants were already uncertain (Arkes & Blumer, 1985). To answer this question, we designed Experiment 2 with a recommendation requesting feature to examine when participants would seek out the algorithm’s response. We expected more requests for the algorithm’s recommendation during the harder trials than the easier trials. Indeed, participants in Experiment 1a and 1b showed an acute proficiency at the easier version of the task giving us little reason to believe they needed the recommendations at all. To narrow our focus onto decision aids themselves, rather than any stimuli-related effects (i.e. a response bias for mammogram judgements in favour of false alarms to missed diagnoses), our subsequent experiments used dot motion stimuli to understand how participants use a recommendation when they voluntarily seek it out.

## Experiment 2: requesting the recommendation

Experiment 2 incorporated two changes to the algorithm. The first was that the algorithm provided recommendations about both outcomes (left- and right-motion). The second change implemented the recommendation request feature. In Experiment 2, participants had the option to request the recommendation on any given trial. One benefit to this request response is that it distinguishes instances when participants did not need the algorithm from instances when they requested but overruled its recommendation.

Alongside these changes, we manipulated block-feedback and training experience. Block-feedback provided a summary of participant performance separately for each difficulty level. We anticipated that performance feedback would prompt performance comparisons with the algorithm and highlight the improvement that comes with selectively requesting the algorithm for the harder images.

Our second manipulation involved training experience. In the previous experiments, participants did not have any training experience with the harder stimuli. In the absence of any error correction during testing, participants may have believed themselves to have discovered a sufficiently workable rule for the harder stimuli and may not have perceived a need to improve their strategy. Introducing training experience with the harder stimuli alongside feedback opportunities should ameliorate any such illusions about their performance.

### Method

#### Participants

Experiment 2 involved 168 psychology undergraduates (*M*_age_ = 19.8, SD = 2.82, female = 109) at UNSW, Sydney. Six participants were excluded (one for failing the instruction check 14 times, five for completing less than half the experiment). The data for a further six participants who did not finish, but completed most of the experiment, were retained for a total of 162 participants. Participants received course credit for participation and were awarded a small payment up to a maximum of $5.00 AUD that was proportional to their performance in the task (*M* = $3.88, SD = $0.22).

#### Materials

##### Stimuli

Experiment 2 used the same random dot motion stimuli from Experiment 1b. We retained the same coherence levels with 0.01 and 0.02 constituting the harder stimuli and 0.20 & 0.25 constituting the easier stimuli.

##### Algorithm

In the training instructions, we introduced the computer algorithm as a 100px-by-100px green box positioned above the stimulus (see example in Fig. 1C). The green box remained on screen until the algorithm was requested or a left/right response was entered. Participants could request the algorithm by pressing the “*g*” key during any trial. The cost of requesting the algorithm was a one-second loading delay. Once requested, a white revolving loading bar rotated within the box (see Fig. 1B). After one second, the green box and loading bar disappeared to reveal a green-coloured arrow pointing either leftward or rightward. The recommendation was independently generated on each trial by randomly drawing a number between one and ten with a 70% probability of displaying the actual correct direction.

##### Algorithm description

Prior to the start of the test stage, we described the algorithm mechanics in more detail. Instructions stated, “The algorithm is there to help you—whenever you see an image, the algorithm will calculate a direction for that very same image”. Just as in the previous experiment we explicitly noted, “There is a 70% chance that [the algorithm] calculates the correct direction. Conversely, there is a 30% chance that it calculates the wrong direction”. Regarding stimulus difficulty, we explained that despite the perceptual difficulty the participant may experience, the algorithm was still able to calculate a direction. We specifically stated, “For both easier and harder images, the algorithm has a 70% chance of calculating the correct direction”.

##### Block of trials

Each block consisted of 80 random dot motion arrays distributed across the difficulty by direction matrix (easier/harder by left-/right-motion). The test stage comprised of six blocks in total and participants were informed of this length.

#### Design

The experiment used a 2 (training) × 2 (feedback) between-subjects design (*n*_min_ = 40). The two levels of the training factor were easy-only (as in Experiment 1a and 1b) and easy-hard training. The easy-only training condition underwent a block of easier training stimuli (80 images) followed by summary feedback. The easy-hard training condition underwent a block of easier training with summary feedback, followed by an additional block of harder images with summary feedback (160 images total). In the results, we examine whether this additional training block led to improvements in motion detection performance.

The training factor was crossed with the feedback factor. The two levels of feedback were block-feedback and no-block-feedback. At the end of a block of images, the block-feedback conditions received a summary screen that stated the (a) proportion correct for easier images, (b) proportion correct for harder images, and (c) overall number of requests for the algorithm in that block. After clicking next, a second screen presented a table with their past performance for easier and harder images in each previous block, including from training.

The no-block-feedback conditions skipped these summary pages and proceeded to a standard “take a break” screen between each block of trials.

#### Procedure

Participants were told their task was to categorize the motion of each stimulus as left- or right-motion. Instructions prior to the training stage explained that an algorithm would be present in training though participants could not interact with it at this point. In training, responses slower than 5 s were given feedback to speed up.

Participants proceeded through their respective training procedures, receiving trial-by-trial feedback following each stimulus. Following a block of training trials, participants also viewed summary feedback for that block. After completing the training stage, further instructions explained the functionality of the algorithm and the subsequent test stage. Participants were briefed about the incentive structure and told there would be six test blocks where the algorithm was available upon request by pressing the “g” key. In the test stage, there was no time limit for an individual trial. Once the test stage was completed, participants were paid proportional to their overall performance up to a maximum of $5.00 AUD.^4^

### Results

We structure the results in the following manner: we first examine participant performance in training and test followed by examining participant algorithm requests.

#### Performance

**Fig. 4:**
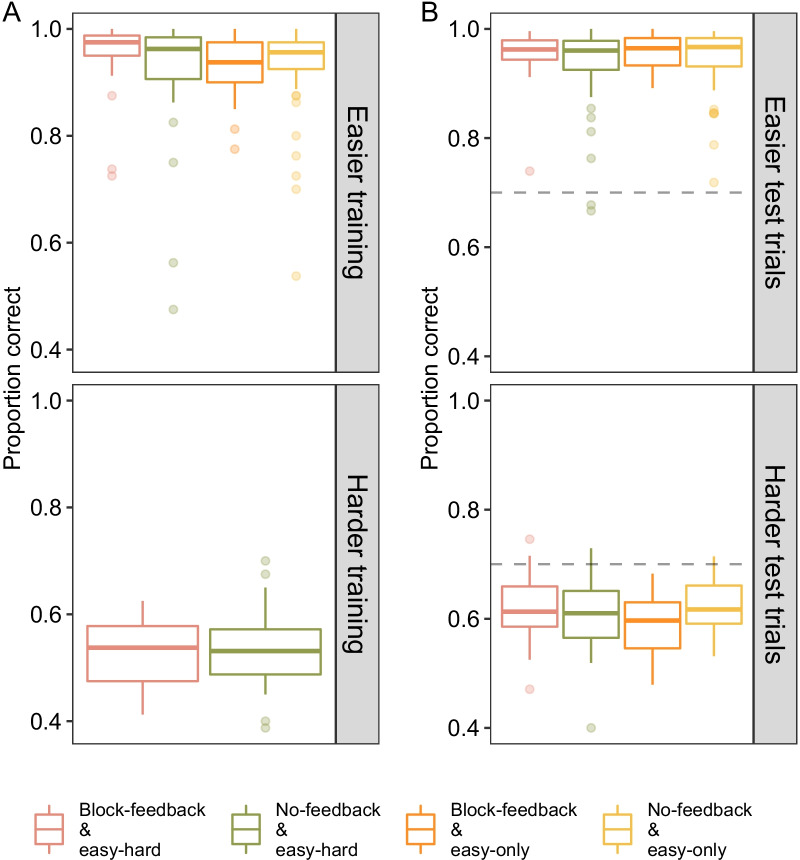
Performance in the experiment for the training blocks (Panel **A**) and test stage (Panel **B**). Mean proportion correct is presented as a function of stimulus difficulty. Boxplots show median and interquartile ranges with dots representing outliers. Note that in training, stimuli were blocked by difficulty, i.e. participants underwent easier training block followed by harder training block in the easy-hard training condition. In test, difficulty was randomized for all conditions. Horizontal line in Panel **B** represents algorithm performance level of 70% correct. See online for colour version

Performance in the training blocks is shown in Panel A of Fig. 4. Performance for the easier stimuli was near-ceiling in both training conditions (*M*_easy-hard_ = 0.94 vs. *M*_easy-only_ = 0.93, SE = 0.01). By comparison, performance for the harder training block was substantially lower and near-chance (*M*_*e*asy-hard_ = 0.53, SE = 0.01). Notably, only a single participant performed as well as the algorithm for these harder trials. For all other participants, we were interested if their relatively low performance in training would lead them to rely on the algorithm during the test stage.

Panel B of Fig. 4 plots performance in the subsequent test stage. Similar to the results in training, stimulus difficulty had a large effect on performance (*F* (1, 158) = 4219. 08, *p* < 0.001, *η*_*p*_^2^ = 0.96). Across all conditions, participants performed better for the easier stimuli (top right, Panel B; *M* = 0.95, SE = 0.01) as compared to the harder stimuli (bottom right, Panel B; *M* = 0.61, SE = 0.01). Interestingly, despite the easy-hard training conditions undergoing an additional training block, levels of performance were similar to the easy-only training conditions that only underwent the easier training block. This suggests that the additional training trials did not improve the detection of motion. Rather, overall improvement in test performance relative to training seems to originate from the degree of algorithm requests.

#### Algorithm requests

**Fig. 5:**
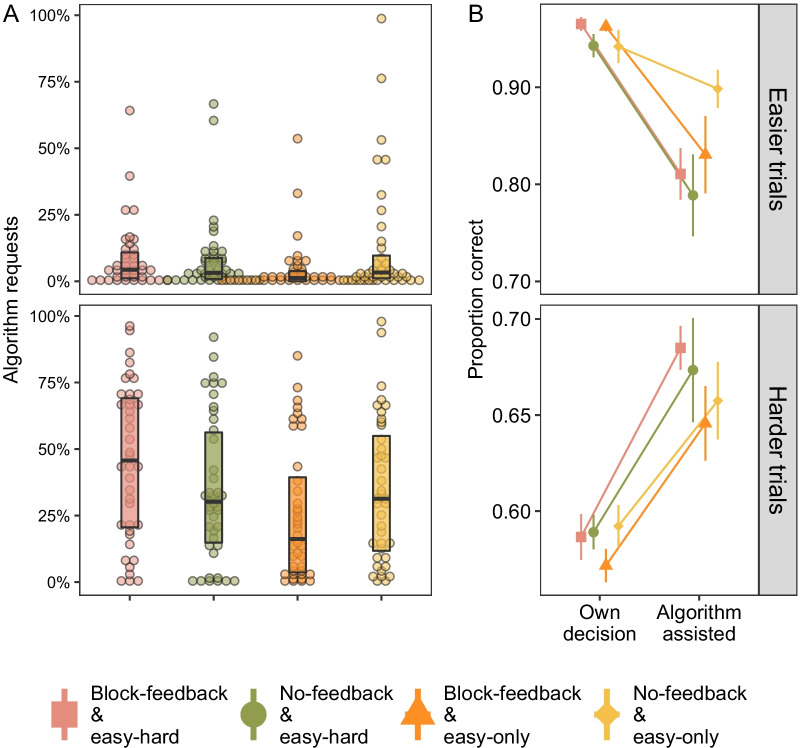
Panel **A** shows mean percentage of algorithm request trials as a function of condition and stimulus difficulty. Boxplot shows the median score and interquartile ranges. Panel **B** shows mean proportion correct with the recommendation on algorithm requested trials compared to unassisted “own decision” trials as a function of stimulus difficulty. See online for colour version

Panel A of Fig. 5 shows the proportion of requests for the algorithm’s recommendation. Across conditions, participants overwhelmingly requested the algorithm when faced with harder stimuli (*F*(1, 158) = 242.56, *p* < 0.001, *η*_*p*_^2^ = 0.61). Most participants made few requests, if any at all, for the easier stimuli (median = 7 requests/240 easier trials). This main effect of difficulty was qualified by a training-by-ease interaction (*F*(1, 158) = 8.61, *p* = 0.004, *η*_*p*_^2^ = 0.05). The interaction speaks to the easy-hard training group requesting the algorithm more than the easy-only training conditions for the harder stimuli (*M*_easy-hard_ = 0.40, *se* = 0.03 vs. *M*_easy-only_ = 0.30, se = 0.03) but not the easier stimuli (*M*_easy-hard_ = 0.08, se = 0.01 vs. *M*_easy-only_ = 0.08, se = 0.02). In other words, training experience increased the subsequent reliance on the algorithm for the appropriate, harder, stimuli.

With regard to feedback, we failed to find any main effects (*F* (1, 158) = 0.25, *p* = 0.62) but, curiously, found a feedback by training interaction (*F*(1, 158) = 5.89, *p* = 0.016; *η*_*p*_^2^ = 0.04). Specifically, we see that for the easy-hard training conditions, requests for the algorithm were numerically higher with block-feedback (first-from-the-left, or red, bar in Fig. 5) than without block-feedback (second, or green, bar in Fig. 5A; averaged over difficulty = 27% vs. 21%, *t*(320) = 1.43, *p* = 0.15). However, for the easy-only training groups, the reverse is true; requests were significantly higher without block-feedback (third, or orange, in Fig. 5A) compared to the block-feedback condition (fourth, or yellow, in Fig. 5; 15% vs. 24%, *t*(320) = 2.16, *p* = 0.03). This result is intriguing because the easy-only training groups did not have any exposure to the harder stimuli before test. While we return to this result in the “Discussion” section, a tentative interpretation may be that summary block-feedback may have encouraged easy-only participants to track their own skill improvement at the harder stimuli rather than emphasize the superiority of the algorithm.

To better understand the relationship between requests and performance, we separated algorithm-assisted test trials from participant’s own decisions in Panel B of Fig. 5. Across all conditions, the algorithm’s recommendation aided performance for the harder trials (lower panel; *M*_own_ = 0.59 vs. *M*_assist_ = 0.67) but decreased performance for the easier trials (*M*_own_ = 0.95 to *M*_assist_ = 0.83). This trial type by difficulty interaction (*F*(1, 130) = 103.78, *p* < 0.001, *η*_*p*_^2^ = 0.44) verified that, indeed, the imperfect algorithm was helpful when participants recruited its recommendation for the harder trials. Despite a slight impairment in performance when requested for the easier trials, the overall low number of requests when the trial was easier shows that participants understood they did not need it.

### Discussion Experiment 2

In Experiment 2, we examined how block-feedback and training affected how people relied on an algorithm’s recommendation. Overall, requests for the algorithm were mostly reserved for the harder stimuli, suggesting that participants distinguished when the recommendation was useful from when it could be misleading. The improvement in performance on algorithm-assisted trials also suggests that participants accepted the recommendation when they asked for it and agreed with its suggestion. We will return to discuss algorithm agreement in the “General discussion” section.

Optimistically, these results suggest that further improvements in performance were possible with requesting the algorithm on more, if not all, harder instances. That is, if participants exclusively relied upon the recommendation for the harder stimuli, they could match the superior performance level of the algorithm. However, even in the condition where we gave the most guidance, by providing block-feedback and training experience, participants fell short of fully capitalizing on this strategy. Why might this be the case?

One motivational explanation is that some participants may have wanted additional practice at the difficult stimuli. Participants likely noticed that some stimuli were considerably more difficult in the task, particularly for the conditions that received easy-hard training. Despite the additional effort involved, however, participants may still have believed they could improve their abilities with additional practice. If there were such motivated individuals in the experiment, then the algorithm may have been treated as a fallback response and only used in cases when participants were completely uncertain. Instead, these individuals may have persisted with the perceptual discrimination elements of the task for far longer under the belief that deferring to the recommendation would rob them of the chance to improve their skills.

This motivational account may also speak to the lack of strong feedback effects. Recall that our motivation for block-feedback was to encourage participants to compare their performance to that of the algorithm. However, an alternative way to use the summary feedback was to track one’s own improvement over time. Following each block, participants may have been more interested in comparing their performance levels to previous blocks rather than to the algorithm. Particularly for the easy-only training conditions that had yet to encounter a harder stimulus, summary feedback between blocks was their only metric to gauge their performance and error-correct their response strategy. Considered together, a speculative interpretation of the lack of feedback effects may be that it reflected different methods to gauge one’s performance; through block-feedback when it was available (forgoing the use of the algorithm), and through requesting the algorithm when feedback was absent.

In the next experiment, we sought to explicitly encourage performance comparisons with the algorithm and strengthen the feedback manipulation.

## Experiment 3: strategy availability

In Experiment 3, our goal was to guide participants towards extensively and *exclusively* relying on the algorithm for the harder stimuli. We drew inspiration from the probability maximization literature and implemented two main changes to encourage algorithm requests as the primary response for harder stimuli.

Our primary manipulation was providing a strategy hint in the instructions. The hint explicitly identified that exclusively relying on the algorithm was the best strategy for the harder stimuli because it would guarantee performance of 70%, akin to probability maximization (Koehler & James, 2010; Newell et al., 2013). If the reason we observed underutilization of the algorithm was that participants did not know about or did not feel licensed to defer their responses entirely to the algorithm, this explicit hint should assuage any such concerns.

Second, we strengthened the block-feedback to additionally include the algorithm’s performance in each block (Shanks et al., 2002). This addition highlighted the fact that the algorithm underperforms on the easier stimuli but outperforms participants for the harder stimuli. We included this additional line to guide comparisons between the participant’s performance and what would happen if they exclusively relied on the algorithm, rather than past performance in previous blocks.

### Method

#### Participants

Experiment 3 was conducted with 67 UNSW undergraduates (*M*_age_ = 19, SD = 1.42, *N*_female_ = 46). Two participants were excluded for completing less than half the experiment. We retained the data for seven participants, who completed all but a small number of trials in the last block, leaving a final *N* of 65. Participants were awarded course credit for participation and a performance bonus of either $0.00, $2.00, or $5.00 AUD, dependent upon their performance (respective *N*’s = 4, 11, and 50).

#### Design

The experiment used a between-subjects design with two conditions. The hint condition received a strategy hint and a performance calculation in the block-feedback summary page, detailed further in the “Materials” section (screenshot Additional file 1: Fig. 6). Prior to start of the test stage, participants in the hint condition received the following instruction page:

“In the previous experiment, we found that most people performed well for the easier images. However, not one person performed higher than 65% correct for the harder images. There is a strategy that will allow anyone to perform better than 65% and finish the task more quickly. *If you use the algorithm selectively for the harder images, you could get 70% of the harder images correct.*”

**Fig. 6:**
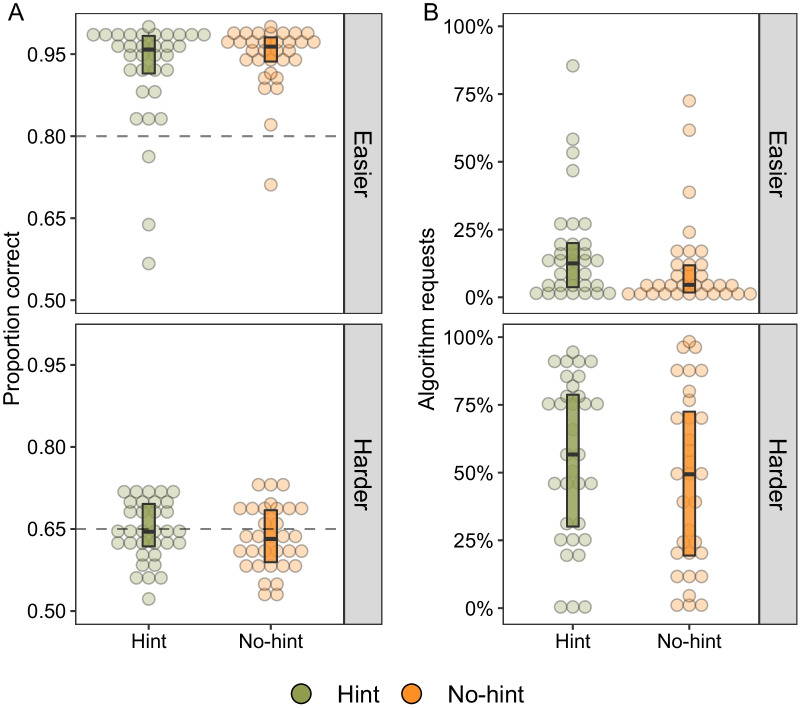
Panel **A** presents the mean proportion correct as a function of difficulty. Intercept lines represent minimum incentive thresholds. An 80% average for easier images awarded $2.00. For the harder images, two out of six blocks of 65% correct awarded the additional $3.00 reward. Panel **B** presents the percentage of algorithm requests. Boxplots display median and interquartile ranges. As a reminder, the green *Hint* condition received a description of maximization and algorithm-performance comparison during block-feedback

The no-hint condition skipped this instruction page and proceeded to the test stage.

#### Materials

##### Algorithm and algorithm description

The algorithm was identical to the one used in Experiment 2. The algorithm description remained the same with only an additional instruction page explaining sampling variability. This page was added to explain why the algorithm’s performance was not exactly 70% in every block but rather would approximate 70% in the long run. We used a coin-toss analogy to illustrate the difference between actual sampling outcomes and the underlying generative process.

##### Block-feedback

In the task, all participants received block-feedback. Recall that during the task, feedback summary screens stated (a) the participant’s overall performance, (b) the number of requests for the algorithm in that block, and (c) a table presenting the performance in past blocks. The table allowed participants to track their progress towards the incentivization goals (screenshot Additional file 1: Figs. 7 and 8).

We made two minor additions to the above; the first was that we also presented the *algorithm’s* performance in addition to the *participant’s* performance. That is, a line stated that “The algorithm got [~]70% of the easier images and [~]70% of the harder images correct” to mirror the participant’s block-feedback (“You got X% of the easier images and Y% of the harder images correct”). The second change was that the hint condition also received an additional statement that calculated the difference between the participant and algorithm’s performance. The line read, “If you had followed the optimal strategy of selecting the algorithm’s response on every harder image, your harder image performance could have been [70%—X] % better/worse”. This line also reminded participants of the maximization strategy.

##### Training

All participants underwent easy-hard training. In Experiment 2, this training manipulation produced the highest proportion of algorithm requests. In conjunction with the strengthened block-feedback in this experiment, we reasoned that easy-hard training would prompt participants to recognize the difficulty of the harder stimuli and defer to the algorithm.

During training, participants received trial-level feedback but could not access the algorithm’s recommendation. That is, after each response in training, participants were told if their response for that stimulus was correct/incorrect. Once the training block concluded, participants were additionally told their overall performance as well as the algorithm’s performance (~ 70% in every block). Note that in the test stages, trial-level feedback was not provided after each stimulus and instead only summary block-feedback was provided at the end of each block.

##### Stimuli

The same random dot stimuli were used from the previous experiments.

##### Incentive structure

Experiment 3 introduced a change to the incentive structure. Prior to the start of the test stage, the instruction page stated that a base bonus of $2.00 AUD would be awarded if participants maintained an average of at least 80% for the easier images across all six blocks. This 80% threshold was chosen to exclude any participant that was blindly following every recommendation, even for the easier stimuli.

If participants qualified for this base bonus, they could earn an additional $3.00 AUD if they performed above 65% in two or more blocks of the harder images. This threshold was chosen because participants in previous experiments could not surpass a 65%-correct threshold without requesting the algorithm on a substantial proportion of the test trials.

Together, changing to these discrete thresholds, from the proportional incentive schemes in previous experiments, emphasized clearer targets for performance with the intent of selectively motivating algorithm requests for the harder stimuli (Fantino & Esfandiari, 2002; Gao & Corter, 2015).

### Procedure

The task procedure was identical to Experiment 2.

### Results

We begin by examining performance in the training stage before the hint conditions diverged. Consistent with the previous experiment, most participants performed well in the easier training block (*M* = 0.91, se = 0.01) but struggled in the harder training block (*M* = 0.52, se = 0.01). We added a novel feature into the training feedback that additionally stated the algorithm’s performance in each training block (*M* = 0.70). We were interested as to whether this feedback encouraged algorithm requests and, in turn, improved test performance.

Figure 6 presents test performance in Panel A. Similar to the training results (not shown), we found a main effect of difficulty, where both conditions performed better for the easier trials (*M*_hint_ = 0.92 and *M*_no-hint_ = 0.95, se = 0.01) than the harder trials (*M*_hint_ = 0.65 and *M*_no-hint_ = 0.63, se = 0.01; *F*(1, 63) = 1086.11, *p* < 0.001, *η*_*p*_^2^ = 0.95). For the easier trials, the vast majority of participants (61/65) outperformed the minimum incentive criteria (shown in the top panel Fig. 6 by horizontal intercept at 80%). However, we did not find evidence of any differences in performance between the strategy hint and no-hint conditions (*F*(1, 63) = 0.198, *p* = 0.65).

**Fig. 7:**
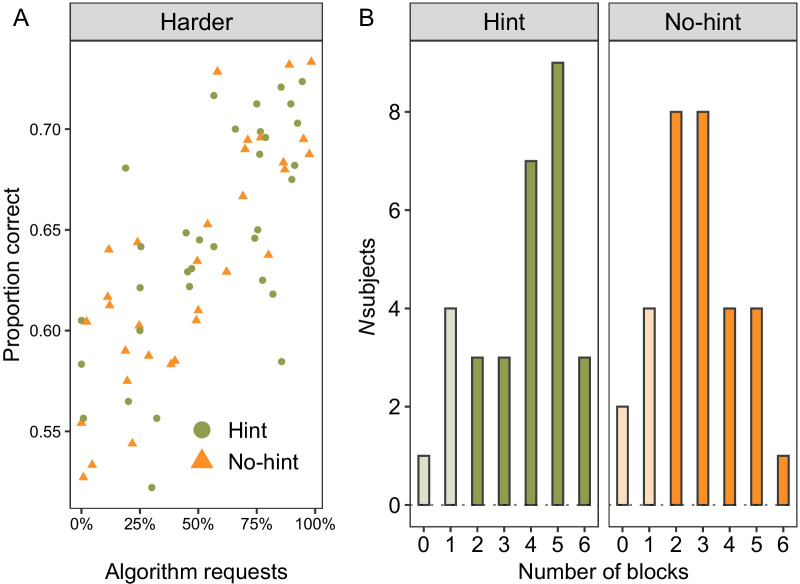
Panel **A** presents the correlation between algorithm requests and performance for the harder stimuli. Panel **B** is a histogram of the number of harder stimuli blocks in which participants reached the high-performance threshold of 65% correct. Darker shading indicates participants that received the additional payment, i.e. reached threshold in at least two blocks, lighter shading indicates no payment

One overall observation for the harder images is that, on average, test performance was higher compared to training performance (*M*_test_ = 0.64 vs. *M*_train_ = 0.52). To better understand this improvement, we plotted the proportion of trials with requests for the algorithm in Panel B of Fig. 6. There were overwhelmingly more requests on the harder trials (*M*_hint_ = 56% and *M*_no-hint_ = 47%) compared to the easier trials (*M*_hint_ = 17% and *M*_no-hint_ = 11%; *F*(1, 63) = 137.74, *p* < 0.001, *η*_*p*_^2^ = 0.69). Participants who made more requests for the algorithm on harder trials also made more correct responses indicating they also agreed with the algorithm’s recommendation (panel A of Fig. 7; *r* = 0.75, *t*(63) = 9.09, *p* < 0.001). However, there is little suggestion that requesting behaviour differed across the hint conditions (*F*(1, 63) = 1.78, *p* = 0.19) or interacted with stimulus difficulty (*F*(1, 63) = 0.21, *p* = 0.65).

The absence of a strong hint effect in the aggregate-level data led us to examine performance at the block level. We were motivated to examine whether the hint conditions differed in obtaining the block-based incentive bonus (Fig. 7, panel B). Presumably, the hint condition possessed an initial advantage because the strategy hint explicitly outlined how to meet incentive threshold from the first block onwards.

Recall that the criterion for the additional bonus was at least *two high-achievement blocks* (i.e. two blocks of harder images > 65% correct). The same number of participants in each condition met this two-block threshold by the end of the experiment (*n*_hint_ = 25/33, *n*_no-hint_ = 25/32, dark shaded bars in Fig. 7, panel B). However, it is interesting to note that the hint condition achieved the high-performance criteria in more blocks overall (*M*_hint_ = 4.2 vs. *M*_no-hint_ = 3.3, *t*(48) = 2.82, *p* = 0.006).

If we consider a higher criterion for the incentive bonus, for instance three or four blocks, there is a numerical advantage in favour of the hint condition (*n* participants meeting three-block criteria; *n*_*hint*_ = 22/33 vs. *n*_no-hint_ = 17/32 and four-block criteria; *n*_*hint*_ = 19/33 vs. *n*_no-hint_ = 9/32). Indeed, these comparisons are post hoc and thus should be treated with appropriate caution. In general, they point towards the idea that participants with the strategy hint more *consistently* met and surpassed the incentive threshold. In the “Discussion” section below, we consider how the strategy hint may have reduced the degree of early exploration of the algorithm within the task.

### Discussion Experiment 3

Experiment 3 incorporated a number of changes that encouraged participants to selectively and extensively use the algorithm. While the main hint manipulation hint did not produce any differences in aggregate-level proportion correct or algorithm requests, we found suggestive evidence that at the block level, the hint condition met the higher-performance threshold in more blocks than the no-hint condition.

While we are cautious of overinterpreting this finding, a preliminary interpretation may be that the no-hint condition engaged in more task exploration in the initial blocks than those in the hint condition. The absence of the hint may have led to greater persistence in testing hypotheses about the usefulness of the cue (e.g. by attempting to second-guess the recommendations). Such experimentation would have left those in the no-hint condition with fewer blocks of trials in which to apply a maximization-like strategy. Indeed, an analysis of algorithm requests across blocks revealed a significant increasing trend in harder-algorithm requests over the experiment while easier-algorithm requests remained stable (stimulus difficulty by block interaction; Greenhouse-Geiser corrected, *F*(3.06, 155.96) = 4.72, *p* = 0.003, *η*_*p*_^2^ = 0.09). Although this trend did not differ by hint condition (*F*(1, 51) = 1.86, *p* = 0.18), it is broadly consistent with the idea that participants learnt that the algorithm was selectively helpful and gradually converged towards relying on its recommendations.

## General discussion

Our experiments aimed to understand better why people use and do not use algorithms. Experiment 1a and 1b demonstrated that people selectively relied upon the algorithm when provided with automatic recommendations. Across two additional experiments, participants who requested the algorithm showed an apt ability to adapt their reliance on the algorithm to suit the situational demands of the task. That is, given feedback about their own abilities and accuracy information about the algorithm, most individuals reserved their algorithm requests for the harder stimuli and ignored or overruled the algorithm’s recommendation for the easier stimuli. This work goes beyond previous investigations of decision-aid reliance by providing individuals with knowledge of the algorithm’s accuracy a priori, opportunities to learn about the algorithm *and* then subsequently test their own judgements against the algorithm’s known level of performance (cf. Arkes et al., 1987). In everyday life, algorithms are espoused for their superior processing capabilities and consequently, decision accuracy compared to human judges. While this superiority may be true, convincing users of their merits may necessitate opportunities to verify and independently test the decision aid’s recommendations against one’s own judgement. Our experiments provided such opportunities alongside a comparison of experimental factors to understand the situations in which people would rely upon a decision aid.

### Learning and verifying the algorithm’s performance

In all our experiments, we explicitly stated that the algorithm was helpful but imperfect. At face value, the algorithm could surpass any individual’s ability for the harder perceptual task, while also making obvious errors for the easier stimuli. Each experimental manipulation was intended to highlight this feature while guiding the participant towards comparing their relative performance against that of the algorithm. However, it might be reasonable for participants to be initially sceptical of our algorithm. In lieu of any experiences with requesting or viewing the algorithm’s recommendations, participants had little evidence to believe that the algorithm would be situationally useful, or even necessary to complete the task.

Viewed in this light, it is interesting to consider what methods participants had to learn and verify the accuracy of the algorithm. One simple verification method was to request a recommendation on an easier trial. One of our most consistent findings was that participant performance on the easier trials was near perfect and remained so across multiple blocks. In other words, participants could be reasonably confident that, for the easier stimuli, they knew the correct response. If requesting was driven by performance comparisons alone, then the time cost of waiting for the recommendation coupled with the algorithm’s errors would make any requesting seemingly wasteful. In spite of this, we observed a non-negligible number of algorithm requests for the easier stimuli in Experiments 2 and even more so in Experiment 3 when our manipulations strengthened the performance comparisons. These behaviours suggest participants may have been strategically requesting these recommendations to learn about the algorithm rather than needing assistance in the perceptual task. With repetition, participants could verify the algorithm’s accuracy against their own experiences without solely relying on the experimenter’s word.

**Fig. 8:**
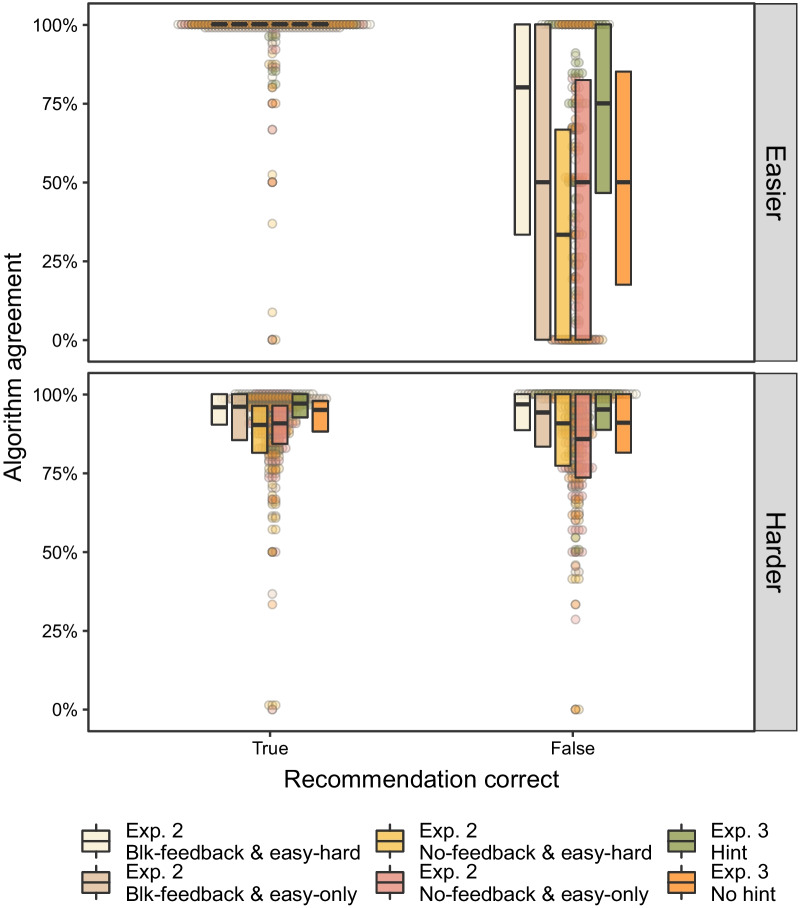
Algorithm agreement plotted as a function of whether the recommendation was correct (*x*-axis) and stimulus difficulty. Data grouped by condition from Experiments 2 and 3. Agreement measures the proportion of trials when the participant made the same response as recommended. Note that only trials in which the recommendation was requested is there agreement data. Boxplots display medians and interquartile ranges with dots displaying individual participant data. See online for colour version

Furthermore, participants were not bound to use the recommendation. Overruling the recommendation was always possible should one feel the algorithm made a mistake. Indeed, data from Experiment 2 and 3 show that participants could aptly determine when to disagree with a recommendation and when to defer to it in lieu of a better response. To illustrate, we plotted data for algorithm agreement in Fig. 8 which measures the proportion of responses when participants agreed with the recommended direction.

Different patterns of algorithm agreement emerge across stimulus difficulty. For the easier images, agreement was near-unanimous when the recommendation was correct (top panel Fig. 8, left bars; median agreement ~ 100%), and lower when the recommendation was incorrect (top panel, right bars). In short, participants could identify the correct response for the easier stimuli and agreed with the algorithm when it was correct. When the algorithm made a mistake, participants *disagreed* with the misleading recommendation.

For the harder images, there was a high-degree of algorithm agreement across recommendation correctness (min. mean agreement = 83%; lower panels of Fig. 8). This suggests that many individuals deferred to the algorithm’s recommendation when they requested the algorithm on a harder trial, consistent with the results showing participants had difficulty with the harder images. These data suggest our manipulations encouraged some participants to adopt a request-and-agree heuristic on harder trials. An interesting nuance is that block-feedback may be central to using such a heuristic. Conditions that did not receive block-feedback seemed more inclined to disagree with the algorithm overall, and potentially more so when the recommendation was incorrect. Broadly speaking, the high degree of agreement across conditions indicates participants learnt when the algorithm was helpful. While they were willing to overrule the algorithm for easier images, the high overall agreement on harder trials combined with the aforementioned increasing requests over blocks (see Additional file 1: Fig. S11 for visualization) shows participants learnt to selectively rely its recommendations.

The verification method may have been most helpful for participants in the early stages of the experiment when they lacked any experiences with the recommendations. However, because of the probabilistic nature of the recommendations, it is likely the algorithm made at least some, and possibly many mistakes, for a subset of participants. One question that arises is whether early poor experiences of the recommendations would shape people’s subsequent beliefs about its usefulness. Early misleading recommendations may lead people to lose confidence in the algorithm’s recommendation capacities and avoid using its recommendations in the future (Dietvorst et al., 2015).

**Fig. 9:**
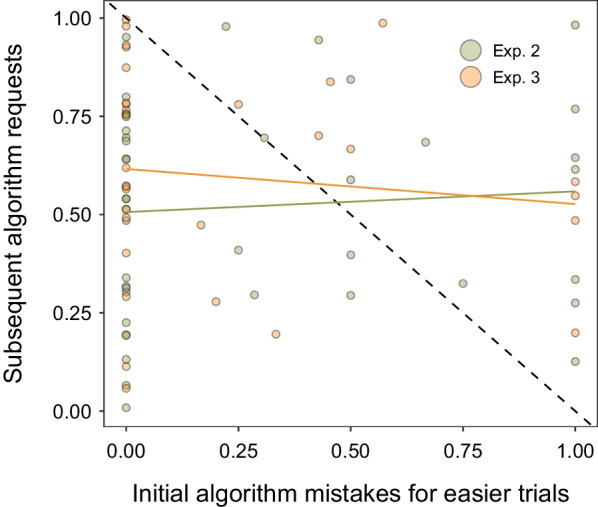
Algorithm requests as a function of initial experiences with the algorithm. *X*-axis plots the proportion of algorithm mistakes for easier stimuli in the first 20 trials of the experiment. *Y*-axis plots the subsequent proportion of algorithm requests for the harder stimuli. Diagonal dotted line illustrates the expected relationship that initially seeing more algorithm mistakes would result in less reliance on the algorithm. Coloured horizontal lines show line of best fit for each experiment. Proportion is plotted on the *x*-axis to account for the different number of trials across participants

To examine how our participants reconciled their early experiences with the algorithm, we examined data for easier requests in the initial test block. Figure 9 plots the proportion of algorithm mistakes as a function of subsequent algorithm requests over the rest of the experiment. We restricted the data to the first 20 trials where participants made at least one algorithm request for an easier trial (*N* = 73; *n*_Exp.2_ = 41, *n*_Exp 3_ = 32). Our intention was to capture the initial learning experiences with the algorithm and show the distribution of early algorithm recommendations, ranging from accurate to wildly errant. We plotted this against the individual’s subsequent reliance on the algorithm for harder stimuli when they may have needed the additional assistance.

From Fig. 9, we can examine whether individuals who experienced more algorithm mistakes would subsequently exhibit the strongest avoidance of the algorithm (shown graphically by the dotted diagonal line). However, this avoidance pattern poorly fits the data. Even for participants that exclusively saw the algorithm make mistakes (i.e. values of 1 on the *x*-axis), the nonzero and wide distribution of subsequent requests suggests that people continued to rely on the algorithm when they needed the help. One explanation for this ongoing reliance is that participants expected the recommendations to be imperfect because the performance information made clear that mistakes were possible.

The expectation for algorithm perfection has been a converging point in recent work examining how people forecast with an algorithm. Dietvorst and Bharti (2020) found decision-makers weight perfect prediction more than overall accuracy when choosing between algorithm-based forecasters. Concurrently, Logg et al. (2019) found that people preferred algorithmic to human forecasts (their own or another person’s) even prior to accruing any experience within the task. This suggests people may initially show *algorithm appreciation* by importing generally positive beliefs about algorithm performance into experiments. Where the forecasting task involves learning and combining multiple variables into a single judgement, individuals may be wise in deferring such complexities to a purpose-designed statistical algorithm. However, when people’s experience of the algorithm unexpectedly reveals that it is an imperfect tool, individuals may subsequently abandon the recommender in what has been termed *algorithm aversion* (Dietvorst et al., 2015, Poursabzi-Sangdeh et al., 2021).

Our experiments contribute to these two accounts by showing how performance information helps set appropriate expectations of the algorithm’s capabilities. Analogous to the difficulty faced in multi-variable forecasting tasks, our participants learnt to appreciate the superior accuracy of the algorithm when faced with the difficult stimuli. When the algorithm made verifiable mistakes for the easier stimuli, the performance information conveyed the expectation that mistakes were possible. Together, we argue it was the performance information that allowed our participants to sidestep the *aversion*/*appreciation* dichotomy and demonstrate flexibility in when they relied upon the algorithm.

### Why not always rely on the algorithm?

Across our experiments, few participants completely deferred to the algorithm. This was the case even in our most guided condition where we explicitly stated that always using the algorithm’s recommendation was the best strategy. However, it is worth remembering that participants, like the decision-makers we intend for them to model, were likely attending to goals other than simply maximizing their rewards. We have previously discussed skill improvement as an intrinsic motivator to persist at the difficult perceptual task. Anecdotally, a number of participants reported a desire to compete against the algorithm. These individuals preferred to rely upon their own abilities and viewed using the recommendations as a crutch. Such a competitive goal would likely suppress the reliance on any decision aid regardless of how helpful its recommendations may have been. Supporting this notion are previous findings where users still overrule and ignore decision aids described to be over 90% accurate (Bartlett & McCarley, 2019; Wiegmann, 2002; Yamani & McCarley, 2018).

The time cost for each recommendation may have also discouraged complete deference to the algorithm. Our motivation for the algorithm’s loading time was to capture the opportunity cost of taking immediate action in favour of seeking more information.^5^ While for an individual trial, the cost was relatively small at only one second, the cumulative costs of always relying on the algorithm may have been prohibitive or at the least, unpalatable.

It is likely that rather than immediately deferring to the algorithm, participants requested a recommendation to avoid spending too long on any given trial. Even with little experience at the task initially, participants may have noticed that some stimuli demanded considerably more effort, and consequently more time to evaluate. If perceptual discrimination was deemed overly effortful, falling back on the algorithm’s recommendation was more likely to yield a correct response than an intuitive but time-consuming guess.

### Broader implications

Decision aids come in various forms, ranging from broad databases searches to highly specific decision trees. Presumably, the utility of a recommendation from these different types of decision aids must also vary; emergency checklists and protocols must be followed whereas a scoping database search could provide several suggestions, only some of which are helpful. To capture this breadth, our experiments incorporated an algorithm with a fixed but imperfect accuracy. However, fixing the accuracy to 70% across stimulus difficulty may have been a suspicious feature of our decision aid, suggesting an inherent flaw in its design. For some participants, the consistent imperfection may have been puzzling and led them to avoid the recommendations all together.

Nevertheless, this constraint on accuracy permitted our experiments to examine a specific question as to how decision-makers adapted their reliance on this decision aid. This question about reliance lies at the heart of applied decision-making. Pilots, firefighters, and doctors need to make judgement calls in uncertain environments. A prespecified algorithm may help cut through the uncertainty but, at its core, the skilled individual needs to weigh any recommendation against their understanding of the problem. How individuals acquire knowledge about the algorithm and any limitations are precisely the kinds of questions suited to laboratory-based investigation where we can examine the underlying capacities that guide behaviours across different environments and decision aids. Even in situations where decision-aids are highly accurate, learning when to trust its recommendations persists as a separate challenge (Dzindolet et al., 2003).

A noteworthy comparison exists in the medical field where decision aids offer a tantalizing promise of improved efficiency and patient care. There is a general sentiment within the medical field that decision aids are useful (e.g. Graham et al., 2003; Ridderikhoff & van Herk, 1999). Yet social, environmental, and behavioural trade-offs exist such that widespread uptake is met with mixed enthusiasm (Longoni et al., 2019). For example, doctors that use decision aids are seen by patients to be less skilled compared to their unaided or even human-aided counterparts (Arkes et al., 2007; Shaffer et al., 2013).

To gauge the application of decision aids in practice, Ridderikhoff and van Herk (1999)^6^ created realistic patient role-plays with an encyclopaedic decision aid for diagnosis problems. The computerized decision aid generated the correct diagnosis in 96% of cases from the very input entered by the participant physicians. However, even when the correct diagnosis topped the recommendation list, most doctors rarely changed their own conclusions and sought information that verified their original hypotheses.

Confirmatory information seeking is not unique to medical decision aids but rather persists as a problem of how individuals consider alternative explanations. Concerns such as perceptions of skill and time pressures may play against decision-makers who seek out and use external sources of information when they are available. Our experiments simplified this broader decision context to retain a focus on the accuracy of the decision aids. Benchmarking the algorithm as the best decision-maker may be useful for identifying when people may be better off outsourcing their cognitive efforts. More specifically encouraging algorithm usage, however, will need to move beyond benchmarks, to understand the competing motivations that affect an expert’s decision to seek additional information.

## Conclusion

The current interest in decision aid algorithms shares a historical thread to the psychological literature on clinical and statistical prediction by Meehl (1954). Although far more sophisticated than in his time, algorithms present a familiar problem for decision-makers as to when it is best to rely upon a statistical tool over one’s own judgement. We explored motivational reasons for why completely deferring to the algorithm was met with resistance but overall, our studies of performance information and decision aids showed that people will rely on the algorithm if they were convinced of its merits. The more informed participants were about the algorithm through feedback and training, the more they relied on the algorithm’s recommendations. Our data therefore provide grounds for optimism that decision-makers can adapt to a world in which algorithms play an increasingly larger role in daily life. Considered together, the experiments paint the decision-maker’s capacity to learn and reason about the algorithm as the cornerstone of how people choose to use decision aids.

## Supplementary Information

**Additional file 1.** For Experiment 1b and all subsequent experiments, we selected coherence levels of 0.25, 0.2, 0.02, and 0.01 where the former two levels were labelled “easier” and the latter two “harder”. The full details of these pilot experiments are presented in Additional file.

## Data Availability

Experimental materials are available upon request. All data and analysis scripts are available at the following link: https://osf.io/47krq/?view_only=6b4539162039449c97d8a44bd8de7c8b.

## Footnotes

1 We use the terms *algorithm* and *decision aid* interchangeably to refer to computational tools that provide decision-makers with a recommendation.

2 A comparison of idealized training with other forms of training was an early focus of our work but has less direct relevance to our current hypotheses. See Additional file 1 for additional details.

3 As a manipulation check, after the training stage a slider-response question asked participants to imagine they were to see a further 100 images and estimate how many images they would categorize correctly. We report these estimates in Additional file 1.

4 As a manipulation check, at the end of the test stage participants were asked to imagine receiving one more image and to state the probability of the algorithm providing the correct response.

5 Separate piloting with an additional monetary cost suppressed but did not eliminate algorithm requests. See Additional file 1 for summary.

6 We thank Hal Arkes for bringing this perplexing result to our attention.
