# Supplementary material for: Adapting to the algorithm: how accuracy comparisons promote the use of a decision aid
Source: Cogn Res Princ Implic. 2022 Feb 8;7:14. doi: 10.1186/s41235-022-00364-y (PMC8825899; doi:10.1186/s41235-022-00364-y)
Supplement: Supplementary file 1 — Additional file 1. For Experiment 1b and all subsequent experiments, we selected coherence levels of 0.25, 0.2, 0.02, and 0.01 where the former two levels were labelled “easier” and the latter two “harder”. The full details of these pilot experiments are presented in Additional file. [file 41235_2022_364_MOESM1_ESM.docx]

**Supplement for the manuscript “Adapting to the algorithm: how accuracy comparisons promote the use of a decision-aid”**

Garston Liang^a^, Jennifer Sloane, Chris Donkin, & Ben Newell

*University of New South Wales, Sydney*

Sections of the Supplement are ordered as they appear in the manuscript with the relevant experiment in parentheses. The sections are as follows:

1. Examples of algorithm and stimuli
2. Difficulty norming experiment for mammogram stimuli (Exp. 1a)
3. Difficulty norming experiment for dot motion stimuli (Exp. 1b)
4. Results for additional training condition (Exp. 1a)
5. Probability matching analysis for Exp. 1a & Exp. 1b
6. Estimates of future performance following training (All exp)
7. Summary of monetary algorithm cost pilot experiment (Exp. 2)
8. Screenshots of task instructions & block feedback (Exp. 3)
9. Trial-level learning curves for algorithm requests (Exp. 2 & 3)

^a^ Corresponding author: Garston Liang, [garston.liang@gmail.com](mailto:garston.liang@gmail.com)

School of Psychology, The University of New South Wales, Sydney,

Kensington, NSW, Australia 2052

1. **Examples of algorithm & stimuli**

Below are examples of the mammogram and dot motion stimuli with their respective versions of the algorithm.


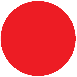

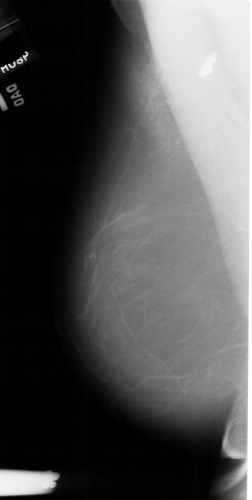

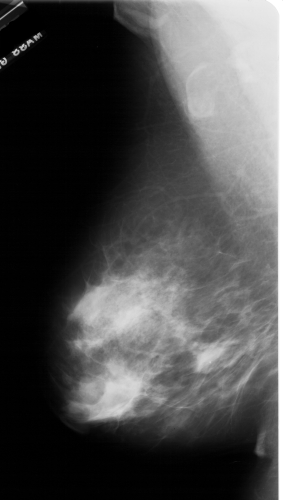

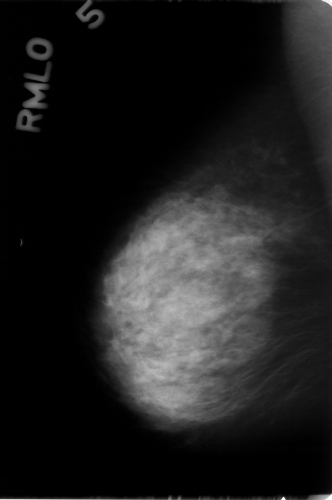


*Figure 1*. Mammogram stimuli used in Experiment 1a with the algorithm’s recommendation of cancer over the middle mammogram image. The left image is of an easier normal stimulus. The right image was an easier cancer stimulus. The middle image is a harder normal image.


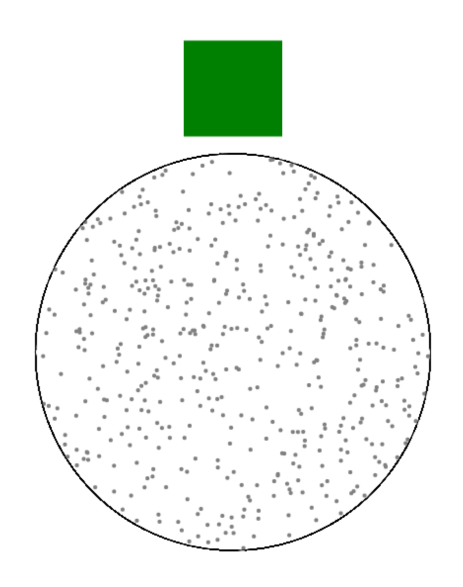


*Figure 2*. Examples of the random dot motion stimuli. Prior to requesting the algorithm, the recommendation was hidden as in the left example. Once requested, the recommendation was revealed after a one second load time as shown in the right example.

1. **Difficulty norming experiment for mammogram stimuli (Exp. 1a)**

We adopted a norming procedure previously used by Hornsby & Love (2014) for their mammogram categorisation experiment. Broadly, this procedure allowed us to determine the difficulty of categorizing any given mammogram image by crowdsourcing the responses of a large number of non-radiologists. With a sufficiently large sample, we could determine the proportion of individuals who intuitively categorised a given image as a *cancer* image. If a large proportion of the sample gave a cancer response and the image in fact contained a tumor, then that image was labelled *easier*. If the responses of the sample were split (i.e. 50% responding *cancer* and 50% responding *normal*), the image difficulty was labelled *harder*. Iteratively repeating this procedure over a large number of images, we could determine the difficulty of our mammogram stimuli for use in the algorithm task.

**Method**

**Participants & materials**. The norming experiment was conducted on MTurk (*N* = 104) under signup constraints that approval ratings were > 90%, English was the primary spoken language, and location based in the US. Participants were paid $6.60 USD for a mean task completion time of 40 minutes. All, but seven participants qualified for the addition $1.00 bonus for above-chance performance. We sourced anonymized mammogram images from the Digital Database for Screening Mammography (DDSM; Heath et al., 2001). All mammograms were presented at the mediolateral oblique (MLO) angle and were selected to be left facing. In cases where the cancer was present in the right breast, right MLO images were horizontally inverted such that the mammogram appeared to be left facing. In total, our sample contained 470 mammogram images comprised of 232 cancer and 238 normal images. Mammograms used in Love and Hornsby (2014), totaling 358 images, were included in our sample as well.

**Procedure.** Participants were introduced to the mammogram task where they were told to provide an intuitive guess as to whether an image was *cancerous* or *normal*. Participants inputted their response on a keyboard by pressing *c* for cancer and *n* for normal. To help with the task, a randomized subset of eight cancer and eight normal mammogram images were provided at the start of each block as an illustrative example. The same examples were always presented so that participants could recalibrate at the start of each block.

The experiment was divided into six blocks with 80 images in each block and fewer images in the final block. Participants did not receive feedback following each image. Participants were given breaks between each block during which the example page was presented on screen. Only once all six blocks were complete, participants received feedback about their overall accuracy.

**Results**

Following our norming procedure, we examined the mammogram images as a function of the proportion of correct participant responses, shown in *Figure* 3. As an example, consider the data for the 0.90 proportion correct cancer images in the left panel. The histogram indicates that two cancer images were correctly identified as cancer by 90% of our MTurk sample. At the opposite extreme, some images contained perceptual features that were misleading. For the 0.10 cancer bin, one cancer image was identified as cancer by only 10% of our sample, and so conversely, 90% of the sample believed the mammogram image to be a normal.


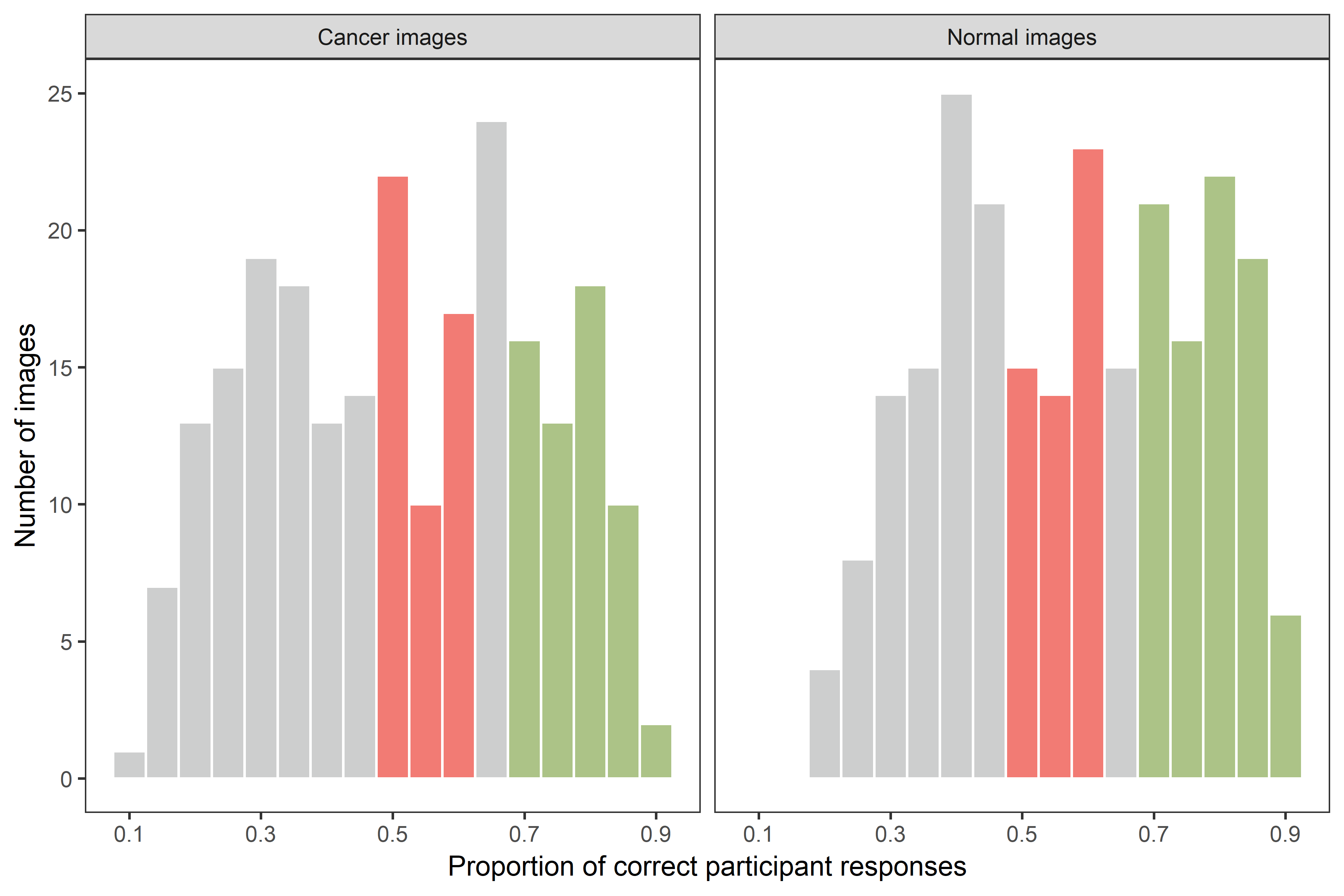


*Figure 3.* Histogram of the number of images binned by the proportion of participant correct responses. Colour indicates the images we retained from the sample while gray bars indicate images that were filtered out. Red bars indicate the number of images that were labelled *harder* and green bars indicate the images labelled *easier*.

From our total sample, we filtered out images below a proportion correct of .50. These images were systematically misleading because most people believed the image to belong to the very opposite category, e.g. the two cancer images described above. With the remaining images, we labelled images that were correctly classified by at least .70 of the sample as *easier*. Images that were correctly classified by at least .50 and at most .65 of our participants were labelled *harder*. After these steps, we retained 267 images (124 cancer & 143 normal) for use in Experiment 1a.

1. **Difficulty norming experiment for dot motion stimuli (Exp. 1b)**

For the dot-motion stimuli, we piloted a number of different coherence levels to determine the difficulty of the motion task. Coherence refers to the proportion of dots moving along the 90˚ - 270˚ horizontal axis. Participants needed to make a response to this proportion with the remaining dots moving along different axes as distractors. Based on our piloting results, we arrived at the final coherence levels of .01, .02, .20, & .25 which provided a clear divide between high performance and chance-performance with the algorithm’s performance situated in between.

**Method**

**Participants & materials.** Undergraduate psychology students (*N* = 34, female = 23, *M*_age_ = 18.7, SD_age_ = 1.7) enrolled at the University of New South Wales, Sydney participated in the study. We used the native dot-motion plugin built for JSPsych (de Leeuw, 2015). We initially selected four coherence levels of .02, .05, .20, and .25 with .20 & .25 as the easier versions. Participants were awarded course participation and a small monetary incentive up to $5.00 AUD that was proportional to their overall performance.

**Procedure.** Participants were introduced to the pilot experiment and the random dot motion stimuli. They were told their task was to respond either left (inputted with the *c* key) or right inputted with the *n* key) based on the direction in which they perceived the dots to be moving. Participants underwent training in which they categorised 80 easier images with trial-level feedback of the correct response. Following the training stage, participants were introduced to the main task in which they received six blocks of trials intermixed with equal proportions of easier, harder, leftward, and rightward motion stimuli. Participants did not receive feedback for the main task until the end of the experiment when their overall accuracy was stated on the final screen alongside an incentive payment.

**Results**

We present the proportion correct for each coherence level in *Figure* 4. Performance for the .20 and .25 coherence levels were at ceiling for most individuals. We retained these two coherence levels and labelled these stimuli *easier* for the main dot motion experiments in the paper. The .02 coherence level proved sufficiently difficult that individuals performed only marginally above chance on average. By comparison, participants responded relatively well to the .05 coherence level and, at least for our purposes, matched the expected performance of the algorithm from the main experiments (.70 in all experiments). Therefore, we opted to drop the .05 coherence level and instead replace it with a .01 coherence level to ensure performance levels were below that of the algorithm. In summary, our piloting results helped us determine that our *easier* stimuli would be the .20 and .25 coherence levels and our *harder* stimuli would use the .01 and .02 coherence levels.


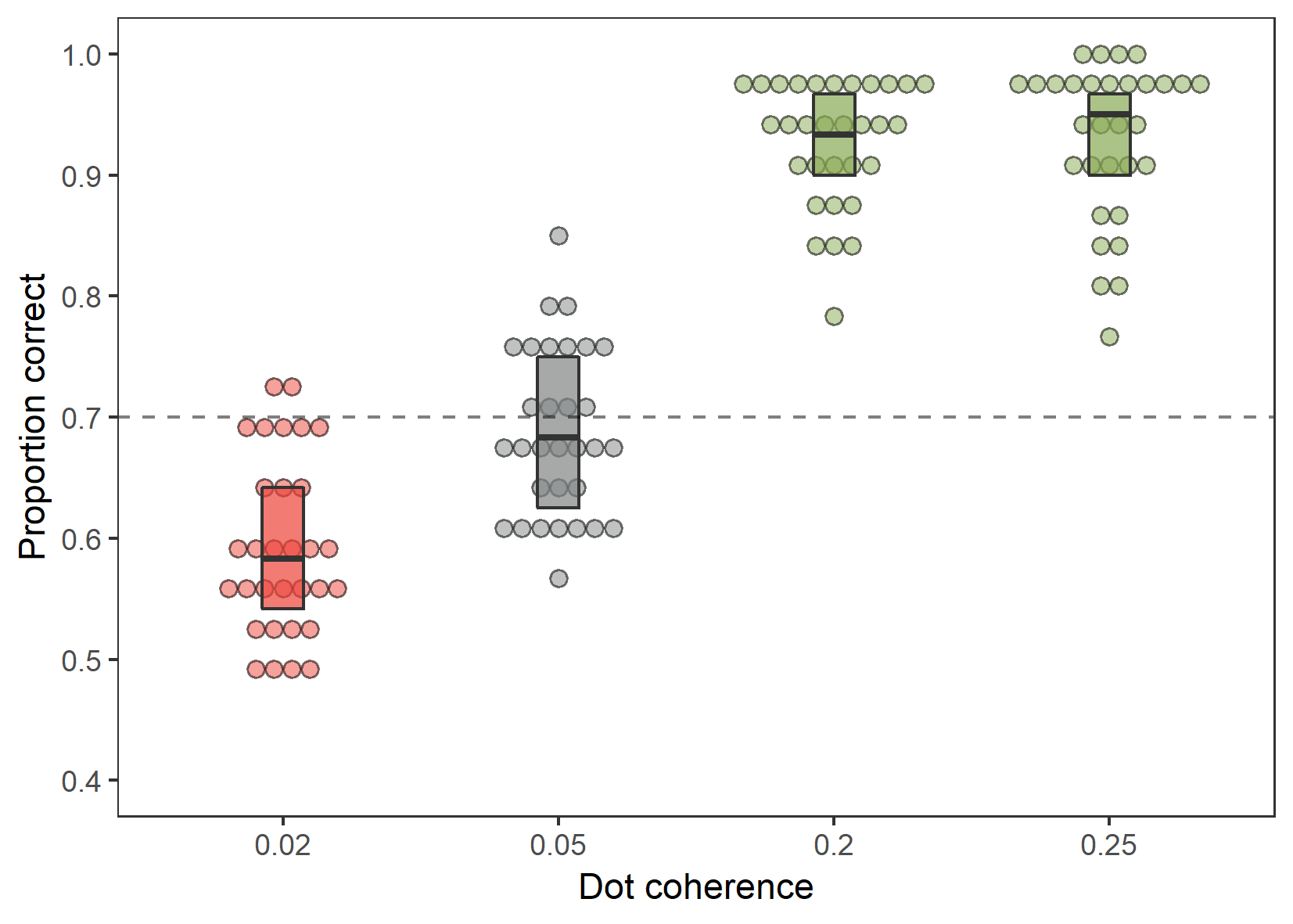


*Figure 4.* Proportion correct as a function of dot coherence. Dotted intercept line represents the performance of the algorithm in Experiment 1b, set at .70. Colour indicates coherence levels that were retained, with green levels labelled *easier* and red levels labelled *harder*. Gray colour indicates the coherence level that was dropped.

1. **Results for additional training condition (Exp. 1a)**

Experiment 1a included an additional training condition that further pursued the line of enquiry into the effects of idealized training manipulation (Hornsby & Love, 2014). This condition received a randomly intermixed block of harder and easier stimuli which differed from the easy-hard condition in Experiment 2 where training images were blocked by difficulty and specifically identified. All other aspects of the procedure remained the same as described in Experiment 1a. Below we present the results for that condition in *Figure* 5 as a function of trial type and stimulus difficulty.


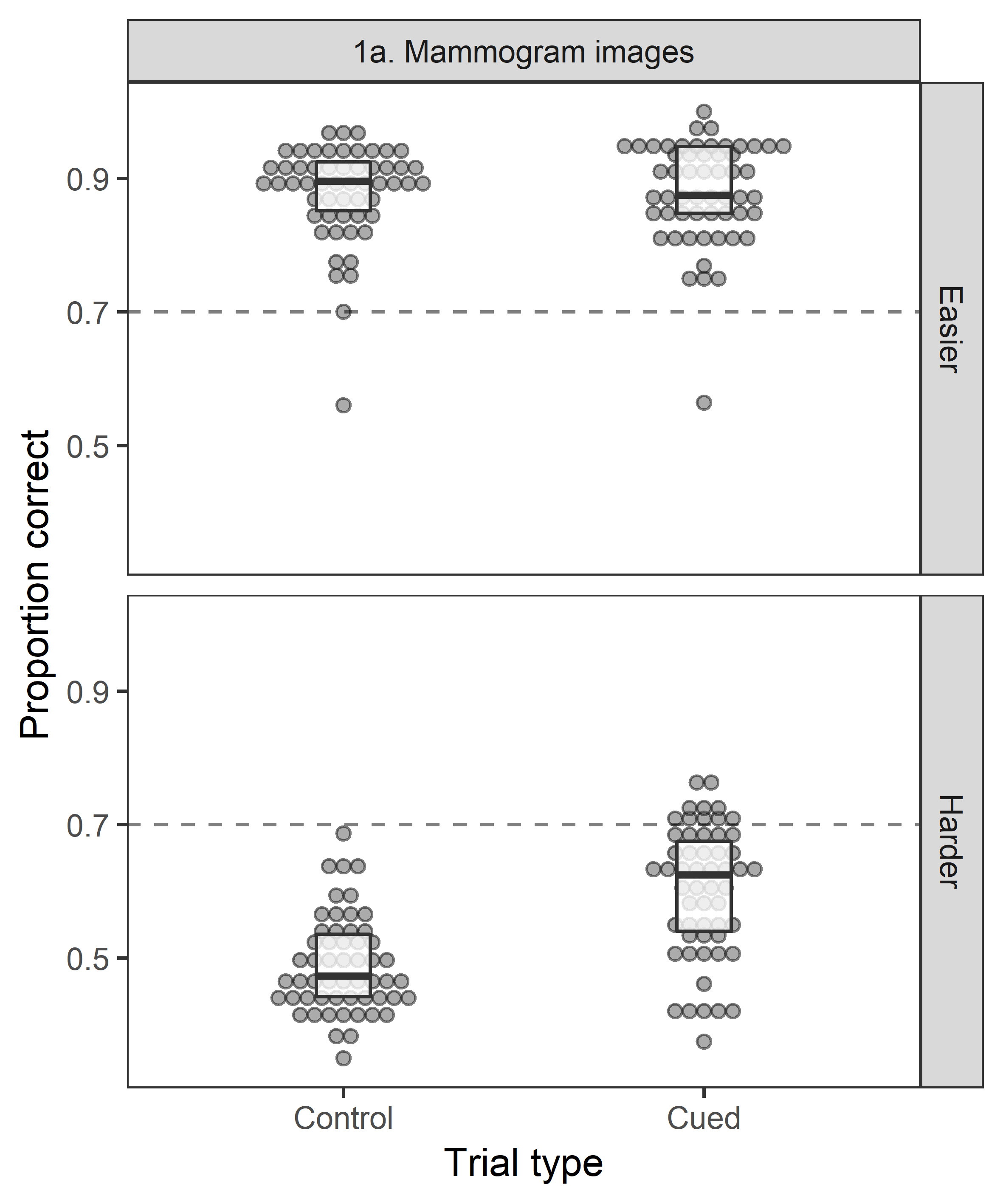


*Figure 5*. Proportion of correct responses as a function of trial type and stimulus difficulty for the additional full-range training condition in Experiment 1a. Dotted intercept line shows the algorithm performance level of .70.

Beginning with the easier trials, most individuals (*N* = 55, female = 34, *M*_age_ = 19.1, SD_age_ = 1.16) performed well across both trial types (*M* = .88). This high level of performance was in spite of the algorithm’s misleading recommendations on 30% of the cued trials, suggesting participants were able to disregard the algorithm on these misleading instances and base their responses on their own perceptual judgements. For the harder trials, performance appears to improve in the presence of the algorithm’s recommendation. While most individuals performed at chance levels in the control trials, mean performance for the cued trials was at .60 (se = .01). This result suggests people were using the automatic recommendation to make a cancer response when it was present.

Taken together, individuals seem to show a selective use of the algorithm’s recommendation. When the stimulus was easier, the algorithm was ignored while if the stimulus was harder, the recommendation was used more frequently. Even in this additional training condition, the degree of algorithm use however, remained below the optimal performance criterion of .70 if one had maximised and agreed with the recommendation on every trial.

1. **Probability matching analysis for Exp. 1a and Exp. 1b**

Probability matching describes a pattern of responses where choice proportions match the probability of an outcome. Applied within this task, probability matching would resemble participants agreeing with the algorithm on 70% of occasions (with a cancer/left response) and disagreeing on 30% of occasions with a normal/right response. Such a strategy would yield an overall proportion correct of 58% (i.e., 70% agree * 70% cue correct + 30% disagree * 30% cue incorrect = 58%). The psychological drivers of probability matching have been extensively investigated (e.g., Schulze & Newell, 2016) though a central role is attributed to the desire to avoid the incorrect recommendation and maintain perfect prediction when outcomes are probabilistic. At least in the aggregate, performance for the harder cued trials were consistent with probability matching (M_1a_ = 0.59, M_1b_ = 0.60; see lower panels of cued trials Figure 2 of main manuscript).

One way to check for probability matching is to examine the proportion of cued responses (i.e., cancer/left responses). If participants used a probability matching strategy, we would expect the proportion of cancer/left response to approximate 70% in the cued trials. Comparing this proportion to the proportion of cued responses in the control block also quantifies the degree of algorithm reliance in the task.

Examining the experiments separately in Figure 6, most participants in Exp. 1a overwhelmingly preferred a cancer response in both the control and cued trials, surpassing the 70% response level of probability matching (shown by the red horizontal intercept). This response bias may reflect the fact that with a cancer diagnosis, the cost of a false alarm is preferable to a missed diagnosis. Participants therefore erred on the side of caution and, at least numerically, made more cancer responses in the presence of the algorithm recommendation. Consistent with this explanation is that this response bias does not appear in Experiment 1b with dot motion arrays.


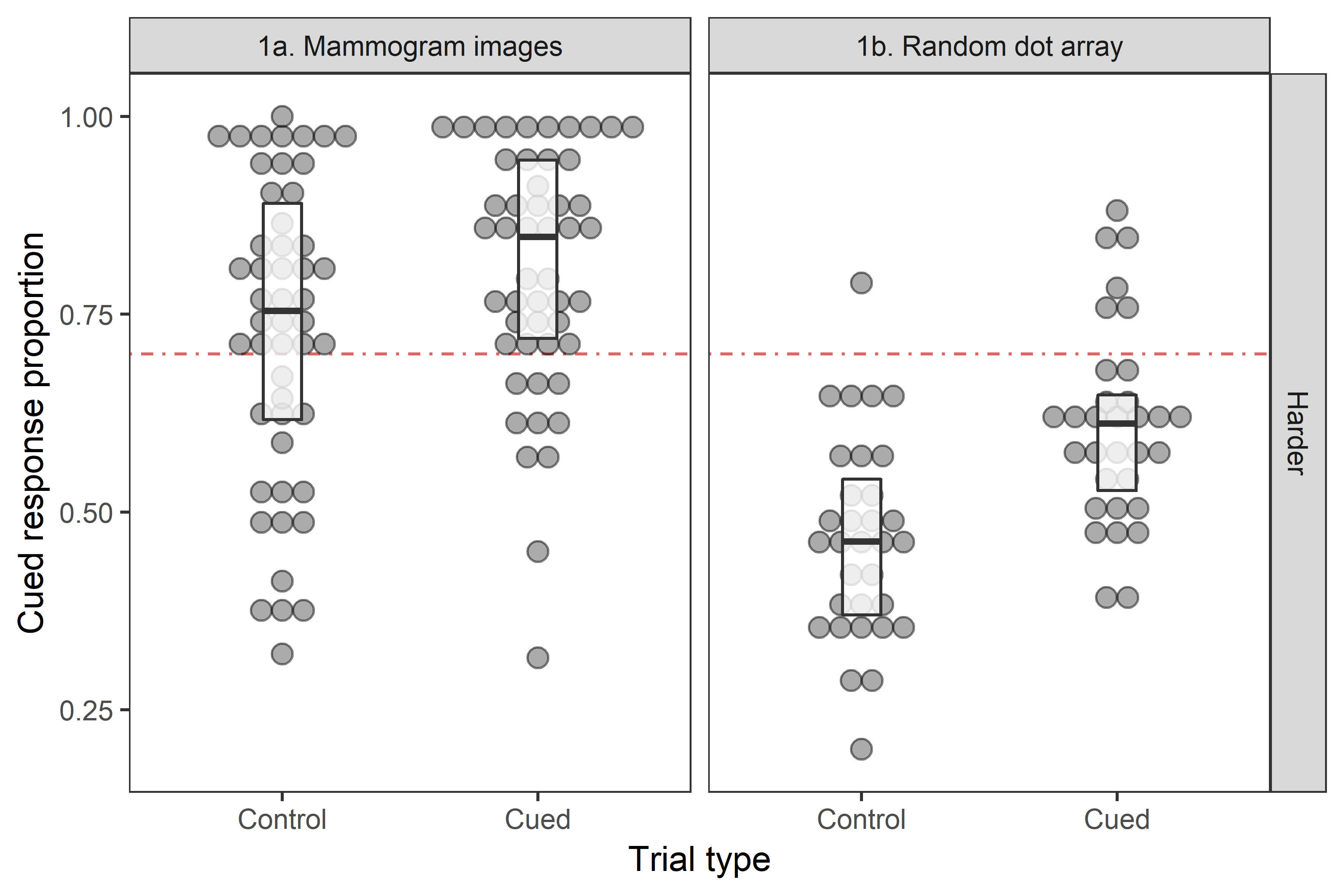


*Figure 6.* Proportion of cued responses (i.e. Exp. 1a response of ‘cancer’; Exp 1b response of ‘left’) as a function of trial type. The red intercept line represents expected cued trial response proportions if probability matching was present, i.e. 70% of the time agree with algorithm, 30% of the time disagree.

In Experiment 1b, the aggregate-level proportion of cued left responses in the control trials is close to 50% indicating participants did not prefer one response over another. When the algorithm’s recommendation was present in the cued trials, participants made an increased number of left responses (*M_cued_* = .61 vs *M_control_* = .46). This increase indicates that people used the algorithm’s recommendation for these harder stimuli. However, participants did not appear to use a probability matching strategy. The proportion of cued responses (*M* = .61) falls short of the prescribed .70. Therefore, despite its appearance at the aggregate-level, probability matching does not explain our results.

1. **Estimates of future performance following training.**

In all experiments, participants proceeded through their respective training procedures, receiving trial-by-trial feedback following each stimulus. After completing a block of training trials, but prior to seeing summary feedback for the training block, participants were asked to imagine they were to receive a further 100 images of the same difficulty and estimate the proportion they would categorise correctly. This measure was a manipulation check on whether participants subjectively experienced the difficulty levels of the stimuli. Figure 7 presents these data for all experiments as a function of training performance and difficulty. If participants were calibrated to their overall performance at the task, their estimates would fall along the diagonal intercept.

Broadly, participants underestimated their future performance though for different reasons across stimulus difficulty. For the easier training blocks where performance was close to ceiling (M = 0.92, se = 0.01), underestimation by most individuals (n = 239/309) appears to be driven by a reluctance to use the extremes of the slider scale. For the harder training block, however, we interpreted the large degree of underestimation (n = 102/147) to reflect the subjective experience of difficulty with the harder images where average performance was at chance-levels (M = 0.53, se = 0.01).

**
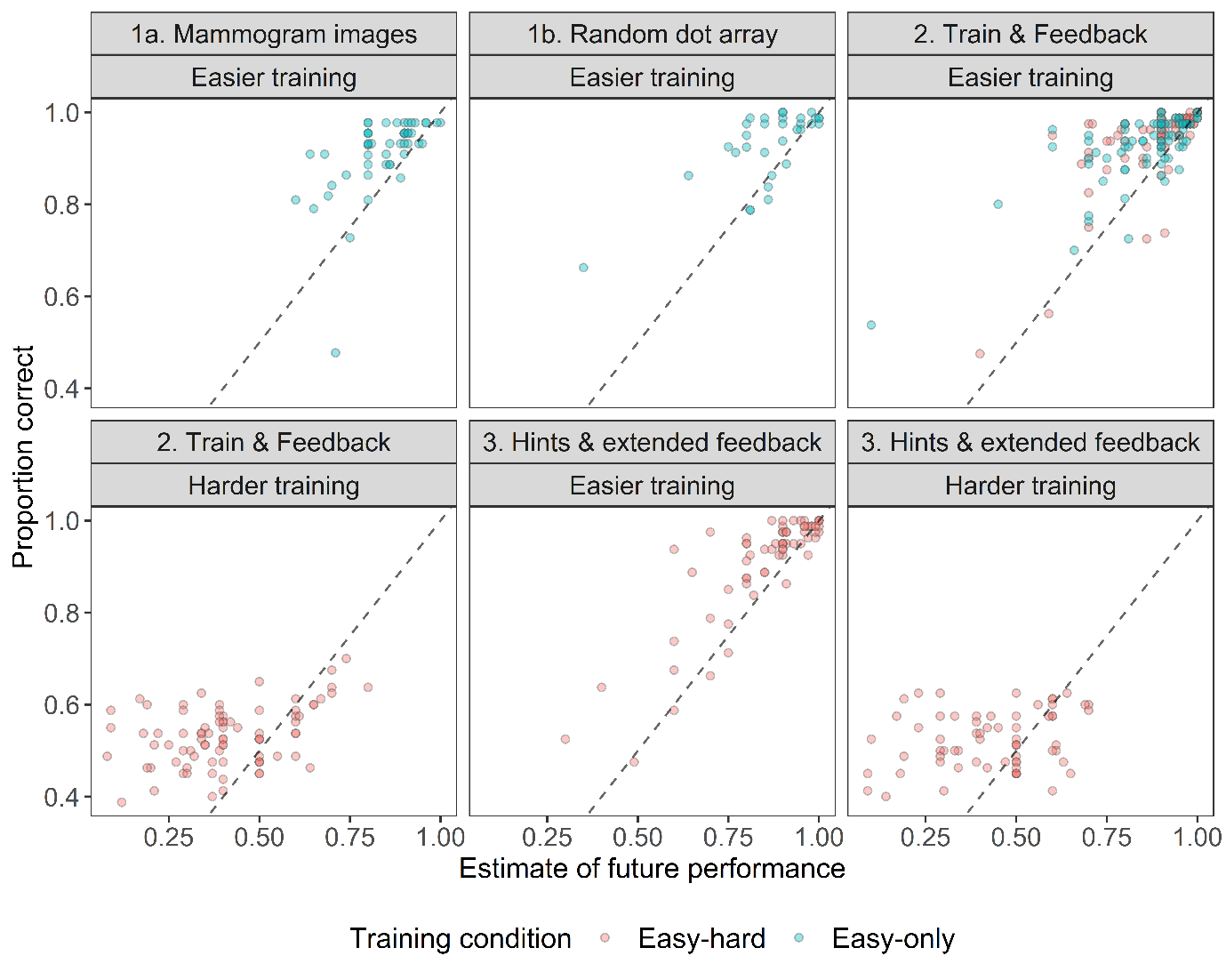
**

*Figure 7.* Proportion of correct responses as a function of estimates for future performance. Panels separately show easier and harder difficulty blocks in each condition, labelled by Experiment number and the primary manipulations. Dotted intercept represents perfect calibration between estimates of future performance and proportion correct. Colour represents training condition. Easy-hard conditions underwent two blocks of training; while easy-only conditions underwent a single easier training block.

1. **Summary of monetary algorithm cost**

In the General Discussion, we explained that our motivation for the algorithm cost being a time-based cost was to capture the opportunity cost of taking immediate action. We also piloted a version (*N* = 44) that included a monetary cost for the algorithm in addition to the one-second load time. In this version, the monetary cost was calculated to be a proportion of the incentive for a single trial.

To elaborate, our monetary incentive in Experiment 2 was paid according to the total accuracy as a proportion of $5.00AUD. This amounted to dividing the total potential payoff, by the total number of test trials (in total 480) and then selecting an appropriate cost proportion. These steps amounted to a cost that was a fraction of a cent per algorithm use. Although calculable given the appropriate knowledge of the task structure, the algorithm cost information may have appeared oddly specific to participants prior to starting the test stage.

We later discovered that our calculation for the algorithm’s cost was set too high – the monetary cost was 70% of an individual trial’s incentive whereas a guess response with a probability of 50% correct would yield a higher expected return. Despite overpricing the algorithm’s recommendation, only 7/44 individuals did not use the algorithm at all. The remaining majority of the participant requested the recommendation at least once and all participants used the algorithm more for the harder stimuli than the easier. In summary, despite being the irrational response, we found that individuals still used the algorithm with a punishing monetary cost.

1. **Screenshots of task instructions & block feedback (Exp. 3)**

The screenshots below illustrate elements within Experiment 3 including a) the hint condition’s maximisation strategy instruction page, and b) the first page of block-feedback containing the hint condition’s comparison line, and c) the table of prior task performance.

Note that except for the cue performance information and comparison line, the block feedback manipulation remained the same as in Experiment 2. These differences between the conditions (hint & no-hint) and experiments (exp. 2 & exp. 3) are noted in the figure captions.

**
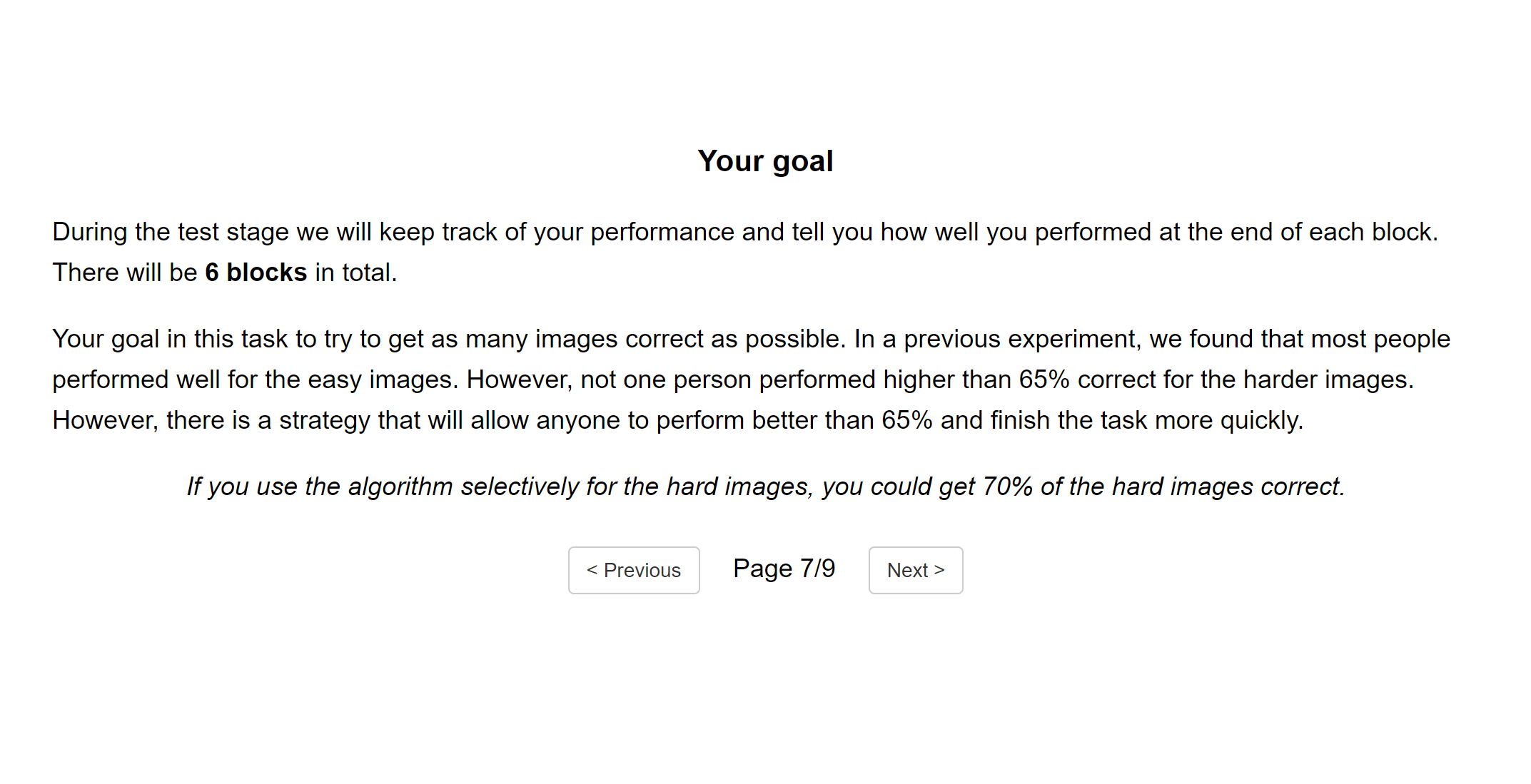
**

*Figure 8*. Instruction page displaying the maximisation strategy hint in Experiment 3. The hint-condition received the instructions inside the orange box. The no-hint condition receives a page with the orange-box instruction omitted.


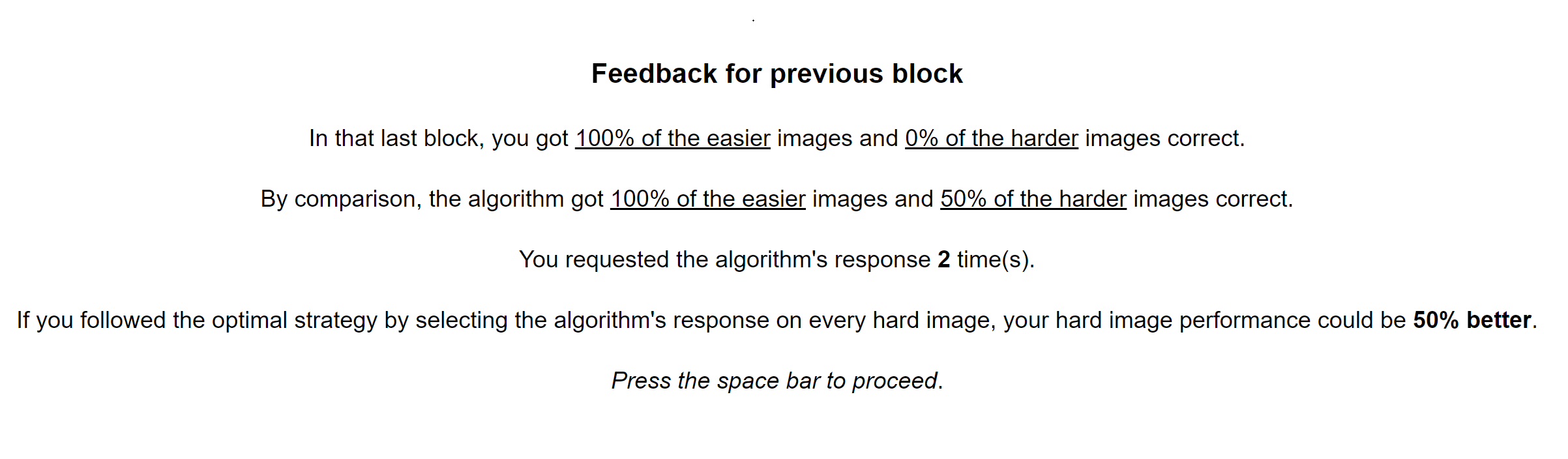


*Figure 9*. Block-feedback page 1 displaying feedback for the previous block’s performance in Experiment 3. The hint-condition’s comparison line is highlighted in orange. The no-hint condition saw the same feedback page with the comparison line omitted. Recall that Experiment 2 used a 2 (training) x 2 (block-feedback) between-subjects design. In Experiment 2, the block-feedback conditions saw a similar feedback screen with the comparison line (orange) and cue-performance information (green) omitted.


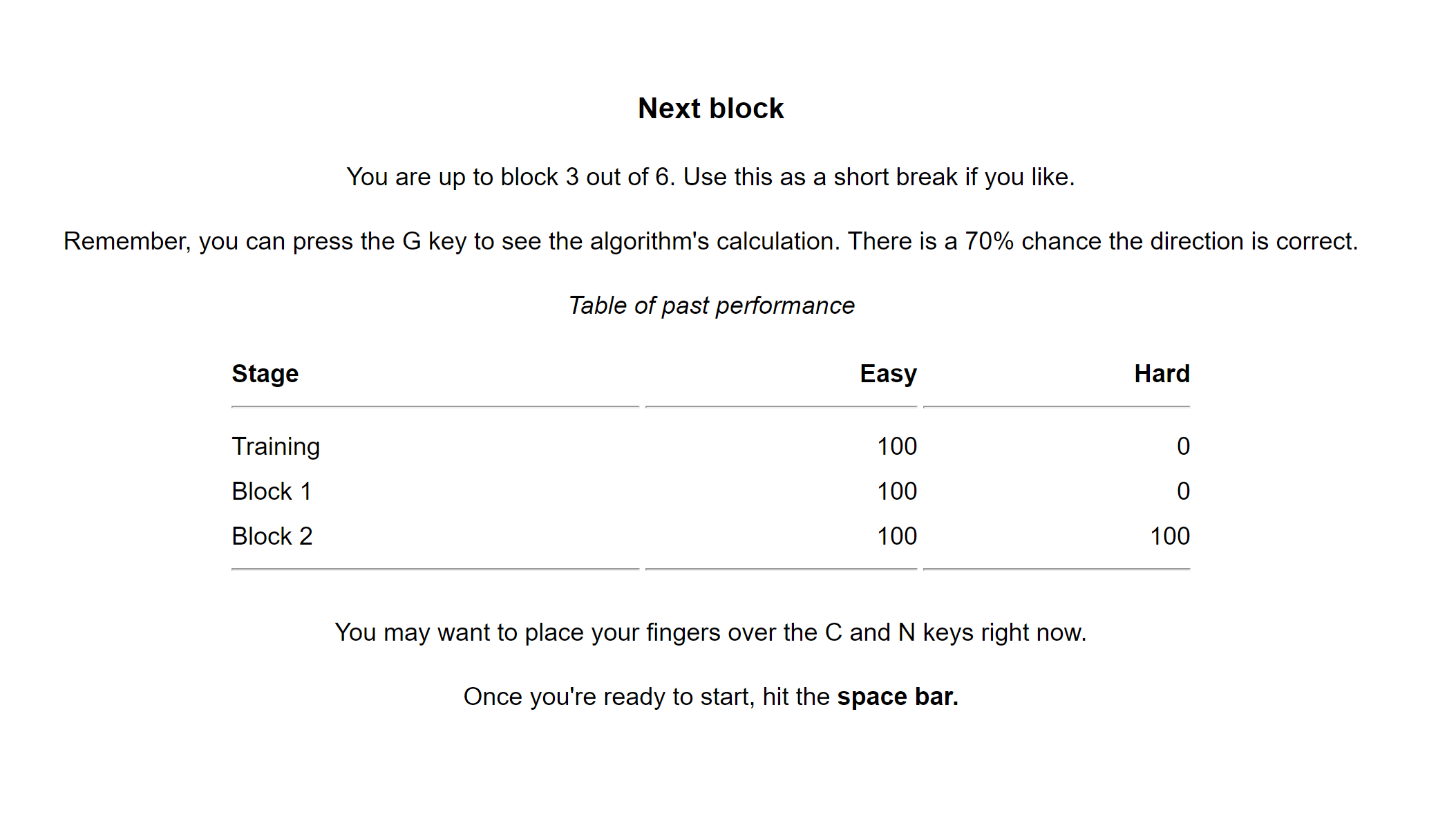


*Figure 10*. Block-feedback page 2 displaying performance over all previous blocks in Experiment 3. This table was updated at the end of each block, displaying past performance broken down by stimulus difficulty. Both conditions (hint and no-hint, Exp. 3) saw this page. In Experiment 2, the block-feedback conditions also received this feedback page.

1. **Trial-level learning curves for algorithm requests (Exp. 2 & 3)**


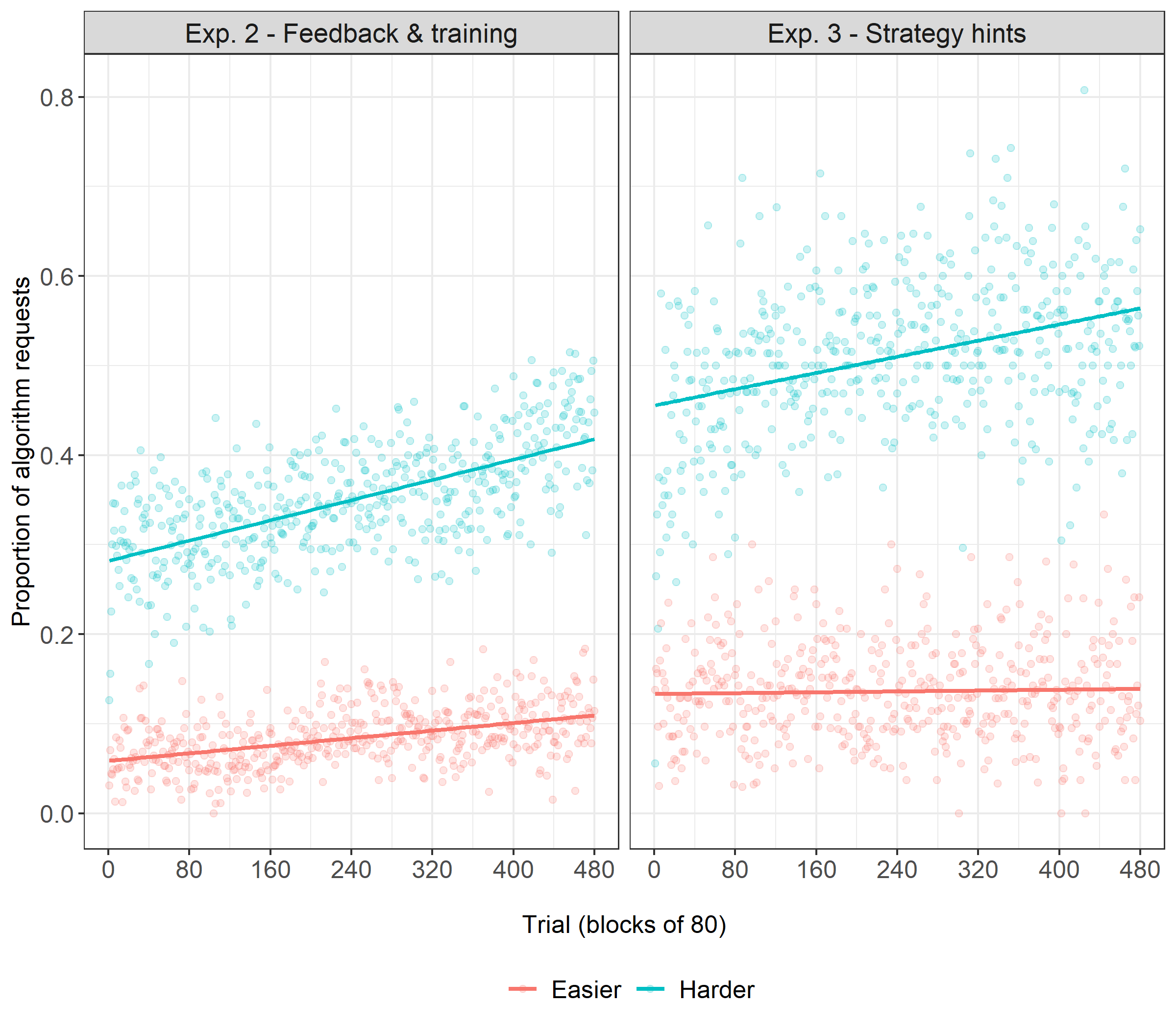


*Figure 11.* Proportion of algorithm requests as a function of trial number and experiment across panels. Regression line shows line of best fit, and dots represent the means for individual trials, averaged over participants. Stimulus difficulty represented in different colours. Note that on each trial, difficulty of the stimulus was randomised for each participant. Consequently, the proportion of easier/harder stimuli on any individual trial along the x-axis is approximately but not precisely 50%.

**References**

De Leeuw, J. R. (2015). jsPsych: A JavaScript library for creating behavioral experiments in a Web browser. *Behavior research methods*, *47*, 1-12.

Heath, M., Bowyer, K., Kopans, D., Moore, R., & Kegelmeyer, P. (2001). The digital database for screening mammography, IWDM-2000.

Hornsby, A. N., & Love, B. C. (2014). Improved classification of mammograms following idealized training. *Journal of applied research in memory and cognition*, *3*, 72-76.

Schulze, C., & Newell, B. R. (2016). Taking the easy way out? Increasing implementation effort reduces probability maximizing under cognitive load. *Memory & Cognition*, *44*, 806-818.
